# Stereoelectronic Features of a Complex Ketene Dimerization Reaction

**DOI:** 10.3390/molecules27010066

**Published:** 2021-12-23

**Authors:** Robert D. Barrows, Mark J. Dresel, Thomas J. Emge, Paul R. Rablen, Spencer Knapp

**Affiliations:** 1 Department of Chemistry and Chemical Biology, Rutgers—The State University of New Jersey, 123 Bevier Road, Piscataway, NJ 08854, USA; rbarrows@fas.harvard.edu (R.D.B.); mjd458@chem.rutgers.edu (M.J.D.); emge@chem.rutgers.edu (T.J.E.); 2 Department of Chemistry and Biochemistry, Swarthmore College, 500 College Avenue, Swarthmore, PA 19081, USA; prablen1@swarthmore.edu

**Keywords:** malaria, amidation, decarboxylation, decarbonylation, carbene insertion, arylketene, Friedel–Crafts

## Abstract

The amidation reaction of a tetrahydroisoquinolin-1-one-4-carboxylic acid is a key step in the multi-kilogram-scale preparation of the antimalarial drug SJ733, now in phase 2 clinical trials. In the course of investigating THIQ carboxamidations, we found that propanephosphonic acid anhydride (T3P) is an effective reagent, although the yield and byproducts vary with the nature and quantity of the base. As a control, the T3P reaction of a 3-(2-thienyl) THIQ was performed in the absence of the amine, and the products were characterized: among them are three dimeric allenes and two dimeric lactones. A nucleophile-promoted ketene dimerization process subject to subtle steric and stereoelectronic effects accounts for their formation. Two novel monomeric products, a decarboxylated isoquinolone and a purple, fused aryl ketone, were also isolated, and mechanisms for their formation from the ketene intermediate are proposed.

## 1. Introduction

Malaria, a debilitating disease spread among humans by the female *Anopheles* mosquito, continues to infect many millions and kills hundreds of thousands. Africa, in particular, was home to 94% of malaria cases and deaths in 2019, and children under the age of 5 accounted for 67% of malaria deaths worldwide [1]. Combination therapies continue to form the first line of drug treatment, but these are encountering resistance among the infecting *Plasmodium* species [2]. The development of new drug candidates with new modes of action has therefore been the subject of wide-ranging and intense research efforts [3].

**Figure 1:**
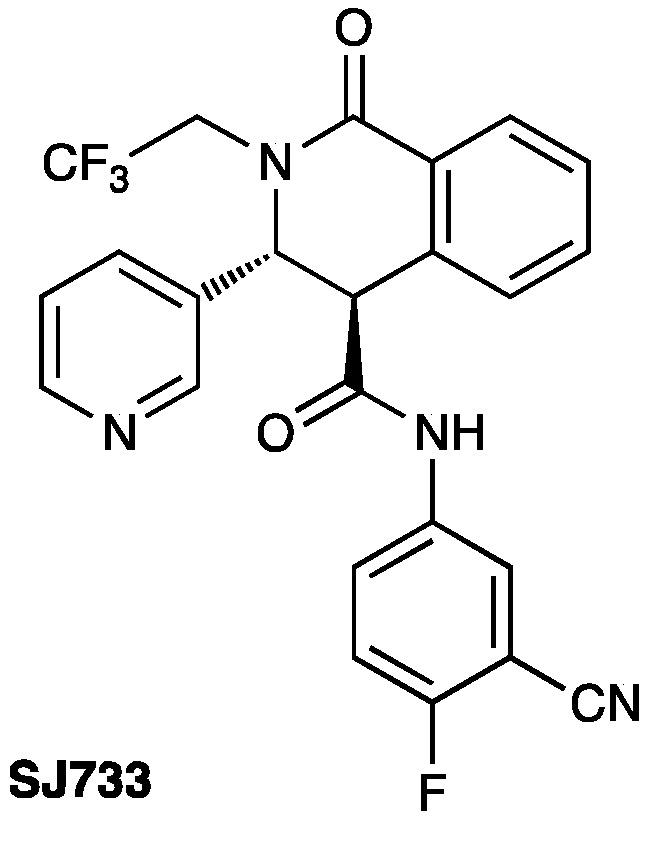
The structure of the new THIQ antimalarial SJ733.

We recently participated in the development of **SJ733** (Figure 1), an ATP4 inhibitor now in phase 2 clinical trials [4,5,6], and also explored improvements in its synthesis [7,8,9,10]. The tetrahydroisoquinolin-1-one (THIQ) 4-carboxanilide core requires an amidation reaction as a key step. In the course of the structure–activity relationship studies on this scaffold [4], hundreds of amides were synthesized, starting from dozens of DHIQ carboxylic acids, including the 3-(2-thienyl) carboxylate, a component of several early hit compounds. Among the more successful reagents to promote this transformation is trimeric propanephosphonic anhydride (T3P). In the course of optimizing the amidation reaction, we studied the T3P reaction of THIQ carboxylates in the absence of the aniline, and found dimeric allenes as the major products [11]. We have now explored this relatively complicated dehydration/dimerization reaction in more detail for the 3-(2-thienyl) example, and report herein the particularly rich range of products, which now include two dimeric lactones, a rearranged, decarbonylated arene, and a purple, tetracyclic ketone. The latter two may be considered unusual for carboxylic acid dehydration reactions. We also offer mechanistic and stereoelectronic rationales for the formation of all products. 

## 2. Results and Discussion

### 2.1. Dimeric Allene Formation from THIQ Carboxylates

**Scheme 1:**
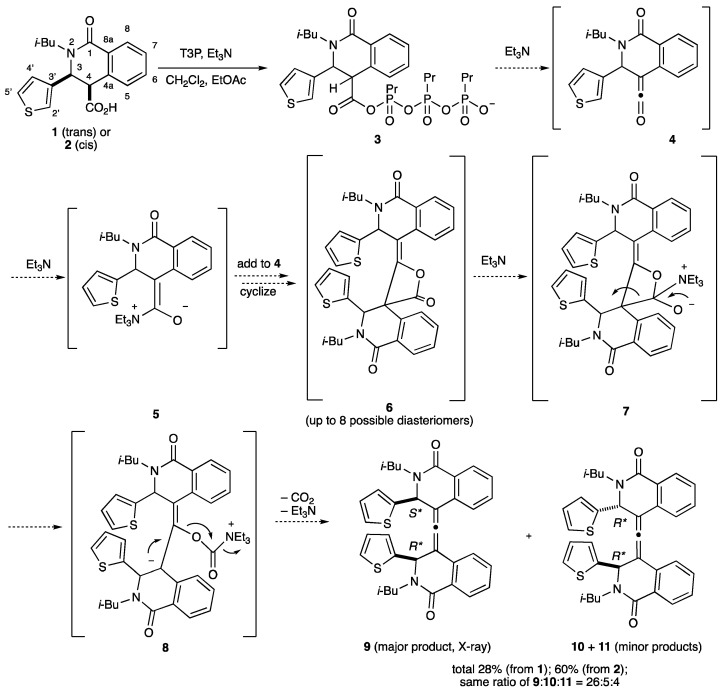
Formation of dimeric allenes from DHIQ carboxylates. The star (*) indicates the relative stereochemistry in a racemic context.

We previously found that treatment of either racemic 3-(2-thienyl) THIQ carboxylate **1** (trans) or **2** (cis) with T3P and triethylamine gave rise to the same isomeric mixture of all three possible dimeric allene products **9**, **10**, and **11** in the yields shown (Scheme 1) [11]. The structure of the major isomer **9** was secured by X-ray crystallographic analysis. The mixed dimeric allene **9** (one enantiomeric component R at C-3 and other S) predominates over the combined R,R/S,S isomers **10** and **11** by only approximately 3:1 (a 1:1 ratio would be stereo-random). A mechanism is proposed for allene formation that takes its major features from the triethylamine-promoted dimerization of tert-butylcyanoketene [12]. Thus, for either carboxylate **1** or **2**, base-promoted formation of the mixed anhydride **3** can be assumed [13,14,15], followed by an elimination reaction to provide chiral ketene **4**. Removal of the relatively acidic H-4 (there is through-conjugation with the C-1 carbonyl group) in T3P reactions of various THIQ carboxylate derivatives can also lead to epimerized carboxamide products [11,16], so this elimination is not unexpected. Indeed, direct evidence for the formation of the ketene **4** was obtained by infrared spectroscopic analysis of crude reaction mixtures in each case [11]. 

In a well-precedented process, nucleophilic addition of triethylamine [17] to the ketene **4** leads to an enolate, which itself can react with a second ketene **4** to provide, after ring closure, a mixture of beta-lactone products **6** (Scheme 1). Because there are three stereogenic centers, as well as an alkene bond that can be E or Z, a total of eight lactone diastereomers is possible, in addition to their enantiomers [11]. Decarboxylation of lactones **6** can be initiated by triethylamine addition to give adduct **7**, followed by ring cleavage to the through-conjugated carbanion **8**. In the *tert*-butylcyanoketene dimerization, stabilization of the intermediate carbanion by cyano can be credited for favoring the opening of the beta-lactone ring [12]. The ability of the THIQ scaffold to stabilize carbanion formation at C-4 [11] can likewise promote the analogous lactone ring-opening process. Loss of triethylamine and carbon dioxide leads to the allenes. The major allene product **9** is a meso-like stereoisomer (there is an internal symmetry element, but also an enantiomer), whereas the other two possible allenes **10** and **11**, which differ in the allene geometry, are diastereomeric [11].

### 2.2. Determination of the Dimeric Lactones

**Figure 2:**
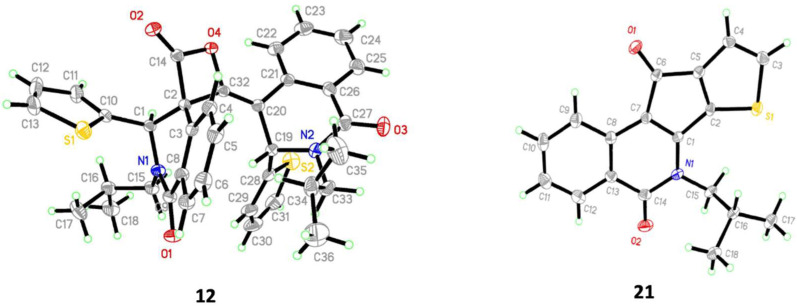
ORTEP representations of the major lactone product (**12**) and the purple ketone product (**21**).

**Scheme 2:**
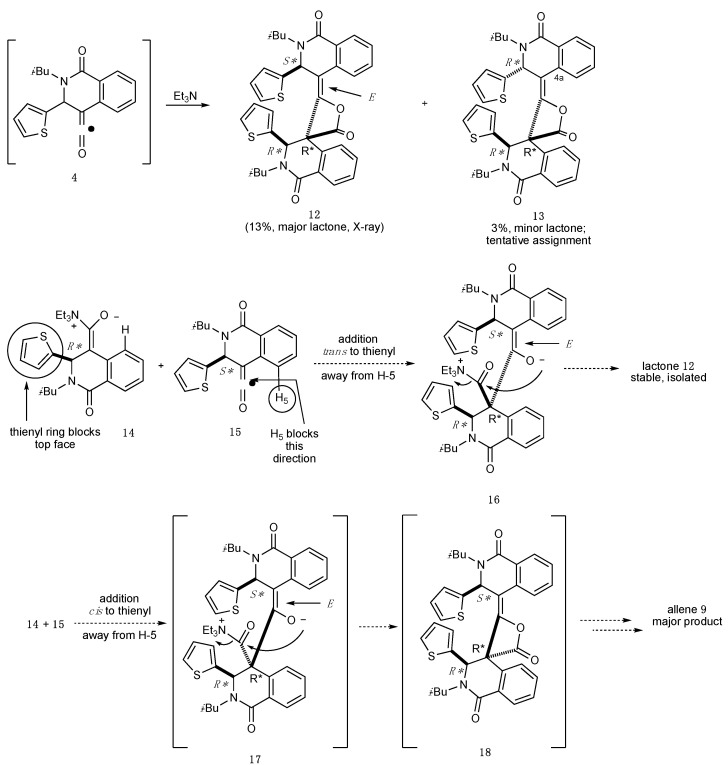
Formation of major dimeric products from ketene **4**. The star (*) indicates the relative stereochemistry in a racemic context.

**Scheme 3:**
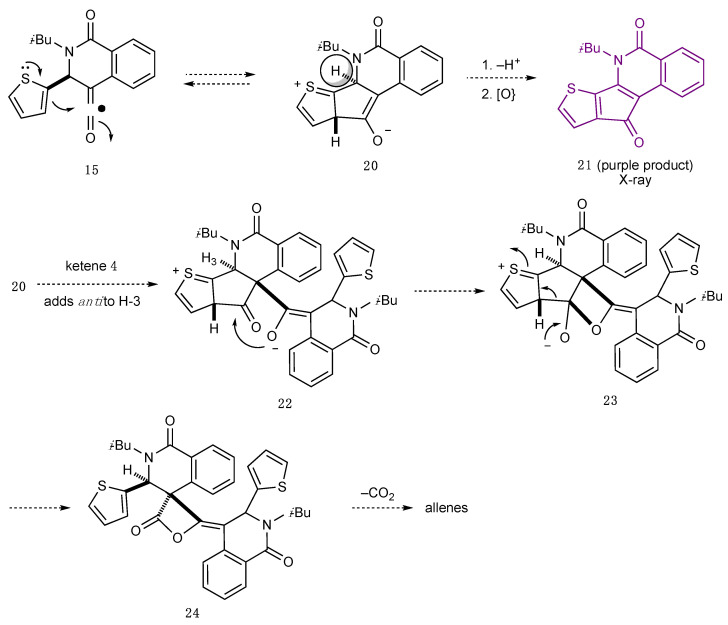
Possible pathway for formation of the purple product, and dimerization pathway leading to allenes.

**Scheme 4:**
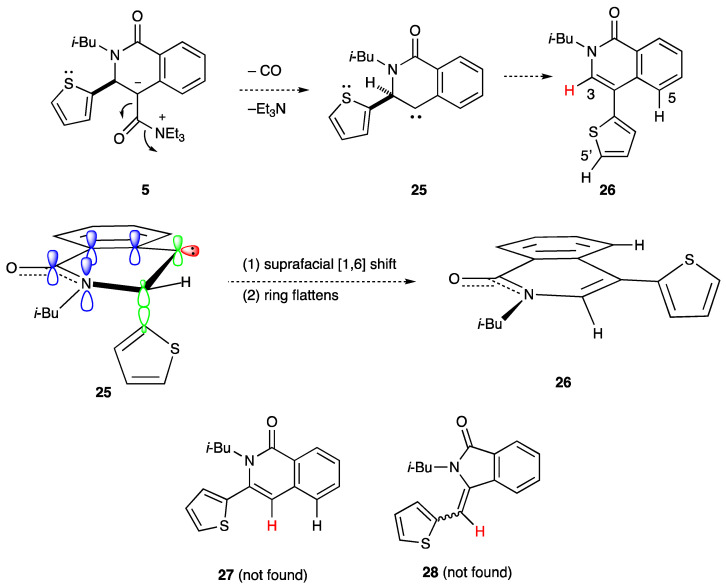
Structure and possible pathway for formation of the decarboxylated product, **26**, and alternative structures ruled out by NMR analysis.

The dimerization reaction of **2** with T3P and triethylamine was repeated, this time with analysis of the crude product mixture by H-1 NMR spectroscopy. An internal NMR integration standard, *cis*-stilbene, was added to quantitate the products. In addition to confirmatory signals for the previously characterized allenes (**9**, **10**, and **11**), evidence was obtained for the presence of two beta-lactones (**12** and **13**), as well as two unexpected products, a fused-ring purple ketone (**21**) and a decarbonylated isoquinolone (**26**). A trace amount of recovered **2** was also detected, as well as the isomerized (trans) THIQ carboxylic acid **1**. The products from this reaction are summarized in Table 1, and structures of **12**, **13**, **21**, and **26** may be found in Scheme 2, Scheme 3 and Scheme 4. All structures are racemic. The details of compound purification and characterization can be found in the Materials and Methods section. ORTEP representations of the crystal structures of major lactone **12** and purple product **21** are shown in Figure 2.

Chromatography of the reaction mixture provided a *beta*-lactone fraction, and the major lactone **12** crystallized from one fraction tube in pure form. X-ray crystallographic analysis revealed this to be the isomer **12**. Subsequent NMR characterization of pure **12** allowed the identification of the diagnostic THIQ H-3 singlets and the lactone C=O signal. Near to these signals in the crude spectrum were found the peaks for one other, minor, *beta*-lactone isomer, tentatively assigned structure **13**. HR-LC-ESI-MS analysis of the crude reaction mixture with selective ion monitoring revealed both major and minor lactone isomers with the required exact mass, and no other dimeric lactone isomers.

Given that eight diastereomers of the dimeric lactone are possible, the formation of only two is remarkable. A rationale for their formation is given in Scheme 2. The analysis begins with the observation that conjugated—that is to say, planar—arylketenes suffer preferential addition by nucleophiles at the carbonyl pi face away from the sterically obstructing ortho-H (here H-5; see **15**) [18,19]. This direction of addition dictates that, in the lactone products following the combination of enolate **14** with ketene **4**, the alkene geometries will be *E*. 

Assuming that the pseudo-axial thienyl group blocks the bottom face of enolate **14**, we might expect ketene **4** to add from above, trans to the thienyl substituent, leading to *E* enolate **16**, and from there to the major *beta*-lactone, **12**. By following the same process, but substituting the R* isomer of ketene **15** (the star (*) indicates the relative stereochemistry in a racemic context), one arrives at an isomeric *beta*-lactone that can provisionally be assigned structure **13**. In other words, combination of the *R* and *S* ketenes leads to **12**, whereas combination of *R* with *R*, or *S* with *S*, would lead to **13**. The “opposite” vs. “same” selectivity would thus be approximately 4:1 for the two ketene enantiomers combining for lactone formation, vs. approximately 3:1 for the allenes.

**Figure 3:**
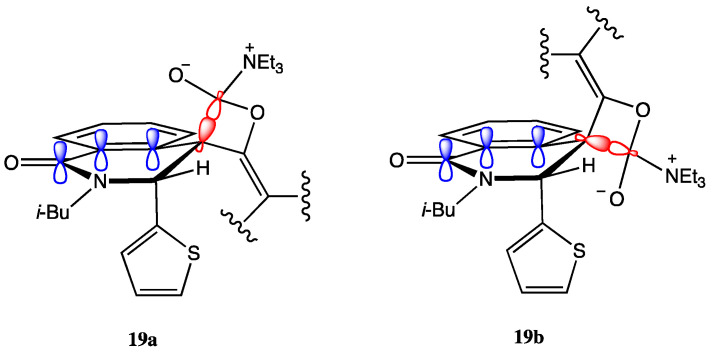
Orbital overlaps in the beta lactone—triethylamine adducts.

The question remains: which lactones might have given rise to the dimeric allene products? The intriguing answer is that the stereoisomeric lactones, e.g., **18**, that result from the addition of ketene **15** to the face of enolate **14** cis to the thienyl substituent might preferentially decarboxylate. A stereoelectronic rationale is provided by the orbital overlaps depicted in **19a** and **19b** (Figure 3). In structure **19a**, the *beta*-lactone/triethylamine adduct from **18** can align its scissile bond (in red) with the pi system of the benzoyl substructure (in blue), and the resulting conjugation may selectively weaken this bond, leading to the allene products. The thienyl substituent ought to remain preferentially in a pseudo-axial disposition. In the epimeric alternative, **19b**, from enolate addition to the ketene trans to the thienyl substituent, the orbital alignment in the triethylamine adduct ought to be less favored. Possibly as a result, lactones with this stereochemistry, such as **12** and **13**, would be more stable, and hence can be isolated.

This notion was explored through calculation of two isomeric structures analogous to **19a** and **19b**, in which the triethylammonium group was replaced with a *tert*-butyl group, the oxetane ring oxygen with a methylene, and the second quinolinone ring (see bond truncations in **18a** and **19b**) with hydrogens. At the B3LYP/6-31G(d) level, the simplified analogue of **19b** proved to be a stable structure, albeit with an elongated scissile bond (1.76 Å). The analogue of **19a**, on the other hand, spontaneously ring-opens, cleaving the scissile bond without barrier. Calculational details are given in the Supplementary Materials.

This analysis raises a second important issue: why might allenes be favored over lactones, by more than 3:1, in the first place? A possible explanation grew out of another set of experimental observations.

### 2.3. Determination of the Other Products

In all of the ketene-forming reactions of **1** and **2**, a purple color developed in the reaction mixture. A purple spot was also visible by TLC analysis, but the amount of purple product was variable and, in all cases, quite small, often 0–1%. However, after one attempted chromatographic separation, a fraction tube containing the purple product deposited crystals over several weeks. X-ray crystallographic analysis revealed this product to be the cyclized and fully conjugated tetracyclic ketone, **21**. Confirmatory MS and NMR spectra were subsequently obtained. The formation of **21** by a cyclization/autooxidation process is proposed in Scheme 3. In this formulation, a Friedel–Crafts-like cyclization of ketene **15** occurs to give enolate **20**, followed by the formal loss of a proton and the C-3 hydrogen as a “hydride”. The oxidant that promotes the conversion of **20** to **21** has not been identified, but it might well be adventitious oxygen.

Participation of the thienyl C-3′, as opposed to the S-1′, in adding to the ketene is supported by ab initio stability calculations on the thienyl-participated intermediates related to **20**. In these studies, it proved easier to examine the *O*-protonated enolates (i.e., the corresponding enols). From ketene **15**, three types of intramolecular nucleophilic addition were considered: attack by thiophene sulfur, attack by thiophene C-3′ on the concave face to yield **20**, and attack by thiophene C-3′ on the convex face to afford the other possible diastereomer of **20** (epimeric at C-3′). At B3LYP/6-31G(d), considering only the lowest energy conformations, *O*-protonated enolate **20** was found to be favored over the isomeric structure formed through sulfur attack by 3.6 kcal/mol, and to be favored over the epimer of protonated **20** by 16.6 kcal/mol. Details are given in the Supplementary Materials.

The possible intervention of enolate **20** suggests an explanation for ketene addition from the ostensibly more hindered face. Because **20** has a flattened structure in comparison to enolate **14**, addition of the second ketene enantiomers can more easily occur from the top face, anti to H-3. In a sense, the internal thienyl nucleophile replaces triethylamine (Scheme 1) as a way to render the ketene nucleophilic. Cyclization to *beta*-lactones **24**, with the release of the thienyl ring, followed by decarboxylation by way of conformation **19a**, provides a route to the allenes (Scheme 3).

A second high-*R_f_* product, which co-chromatographs with ketone **21** in many eluants, was identified in the mixture as the decarboxylated isoquinolin-1-one, **26** (Scheme 4), and then was partially purified. The structure is assigned to **26** based on its mass, H-1 and C-13 spectra, and by comparison to reference compounds in the literature. In particular, the new ring methine H-3 (in red) is shifted downfield (7.17 ppm; compare 7.20 ppm reported for the closely related N-methyl analogue [20]) relative to a possible isomeric structure with thienyl at C-3 (**27**). The latter compound (H-4, in red, expected at 6.6 ppm [21]), which would have resulted from a C-3 to C-4 hydride shift, was not detected in the reaction of **2**. Likewise, a ring-contracted indolinone **28** can be ruled out (and was not detected as a product) based on the absence of the 1 H (in red) singlet expected at 6.7–6.8 ppm [22,23]. The 2′ attachment of the thienyl ring is retained in **26**, as confirmed by the thienyl coupling pattern (e.g., H-5′, dd, *J* = 4.8, 1.6 Hz). 

A possible mechanism for the formation of **26** is presented in Scheme 4. The triethylamine adduct, enolate **5**, upon encountering no ketene **4** remaining for addition, can decarbonylate with loss of triethylamine to generate singlet carbene **25**. A formal suprafacial six-electron [1,6] shift of the thienyl ring to the empty pi orbital on the bottom face of preferred conformation **25** leads to **26**. An orbital picture of this event is depicted in Scheme 4. Photochemical and metal-mediated decarbonylations of ketenes under mild conditions are well-known, whereas thermal examples usually require high temperatures [24,25,26]. In the case of **5**, room-temperature loss of CO may be driven by the stability of the resulting through-conjugated carbene **25**. Carbene insertion into the C-C bond of adjacent electron-rich aryl substituents has been previously reported, as has vicinal 2-thienyl migration in a gold–carbene intermediate [27,28].

Finally, we offer an explanation for the formation of trans acid **1** among the products of the dehydration of cis acid **2**. One-way triethylamine-promoted partial epimerization of mixed anhydride **3** (Scheme 1) to the more stable trans isomer was previously implicated in the formation of trans amide products from **2 [11]**. It is reasonable for this process to also occur in the absence of an amine nucleophile. The trans isomer of **3** should (and does) eliminate more slowly than the cis because the removable H-4 of the former is predominantly pseudo-equatorial and its C-H sigma bond is relatively more poorly conjugated with the benzoyl substructure.

## 3. Conclusions

The eight reaction products (Table 1) of the T3P-promoted dehydration of the chiral THIQ carboxylic acid **2** are unusually varied and their formation is rich in mechanistic and stereochemical detail. Seven of them are accounted for by initial ketene production (see **4**). The eighth (**1**) derives from the epimerization product of mixed anhydride intermediate **3**. This process was suggested previously [11] and is now confirmed. Pathways leading to the formation of dimeric lactones **12** and **13**, and dimeric allenes **9**–**11**, have a literature precedent in the reactions of unrelated ketenes. However, the tetracyclic ketone **21** that results from an intramolecular Friedel–Crafts-type cyclization and dehydrogenation appears to represent a new ketene process [29]. Likewise, room-temperature decarbonylation of **4**, leading to the rearranged arene ketone **26**, is without apparent direct precedent [29]. The complex structure of the starting material (**2**) permits a mechanism-based understanding of the steric and stereochemical details of product formation. The proximity of the electron-rich 3-(2-thienyl) ring of **2** to the ketene unit at C-4 undoubtably fosters the new ketene pathways.

## 4. Materials and Methods

### 4.1. General

Organic solvents used for reactions were of reagent grade and were used as received. Gravity chromatography was performed by using silica gel (Sorbent technologies 230–400 mesh) as the stationary phase. Silica gel 60 F_254_ pre-coated plates were used for thin-layer chromatography, and visualization was accomplished with UV light (254 nm) or ninhydrin stain. The LC-MS and high-resolution mass spectrometry (HRMS) analyses were conducted using a Xevo G2-XS QTOF instrument (Waters Corporation, Milford, MA, USA) with electrospray ionization. The LC-MS samples were separated on a Acquity UPLC (Waters Corporation, Milford, MA, USA) C18 1.7 μm column as solutions in water or acetonitrile, prepared at 0.15–0.20 mg/mL concentration. The LC-MS chromatography was carried out with linear gradients of 0.05% formic acid in acetonitrile and 0.05% formic acid water. ^1^H, ^13^C, and HSQC NMR spectra were obtained on a Varian VNMRS 500 or 400 instrument or a Bruker Avance Neo 500 (Bruker, San Jose, CA, USA). Chemical shifts (δ) are reported in parts per million (ppm) and are referenced to the residual solvent signal. Coupling constants (*J*) are reported in hertz (Hz). The usual abbreviations are used to describe multiplicities: s (singlet), d (doublet), t (triplet), q (quartet), br (broad), app (apparent). NMR solvents and all other commercially available reagents were used as received and without any further purification. Trace impurities such as solvents, water, and grease are not reported in the ^1^H-NMR transcription.

### 4.2. Dehydration/Dimerization of (3S*,4R*)-2-Isobutyl-1-oxo-3-(thiophen-3-yl)-1,2,3,4-tetrahydroisoquinoline-4-carboxylic Acid (***4***)

A solution of *cis* carboxylic acid **4** (92 mg, 0.287 mmol) and triethylamine (160 microliters, 1.14 mmol, 4 equiv) in dichloromethane (5 mL) was stirred at 0 °C. A commercial 50% solution of *n*-propanephosphonic acid anhydride (T3P) in ethyl acetate (682 microliters, 1.14 mmol, 4 equiv) was added dropwise. The resulting solution was stirred at 0 °C for 10 min, and then was allowed to warm to room temperature and stirred for 16 h. The reaction mixture was concentrated, and the residue was partitioned between ethyl acetate (25 mL) and pH 7 phosphate-buffered saline (PBS buffer, 25 mL). The organic layer was concentrated to a residue that was analyzed by H-1 NMR spectroscopy with *cis*-stilbene as internal integration standard (δ 6.57, s, 2H) to ascertain crude yields. Integration of the well-resolved tetrahydroquinoline H-3 signals between 4.9 and 5.7 ppm was used for this determination, along with the respective signals at 7.37 ppm for **21** and 7.17 ppm for **27**. The identities and amounts of products in the mixture are given in Table 1. The crude product was column-chromatographed on silica gel with 10:1 hexane/ethyl acetate as the eluant to afford the high *R_f_* mixture (H-1 NMR analysis) containing purple product **21** and decarboxylated product **26** as a film, *R_f_* = 0.42. The mixture of **21** and **26** was re-chromatographed with 1:3 toluene/dichloromethane as the eluant to afford partially purified **21**, *R_f_* = 0.60, 2 mg, and **26**, *R_f_* = 0.25, 4 mg. One of the chromatography fractions deposited **21** as purple crystals, mp 194–195 °C, that were suitable for X-ray crystallographic analysis. Fractions containing the three dimeric allene products **9**, **10**, and **11** were combined (*R_f_* = 0.31, 4:1 hexane/ethyl acetate), analyzed by H-1 NMR spectroscopy as before [11], and their identities and ratios were confirmed. Fractions containing the lactones **12** and **13** (*R_f_* = 0.28, 4:1 hexane/ethyl acetate) were collected. Crystals of **12** suitable for X-ray crystallographic analysis, mp 255.0–255.8 °C, formed from one fraction, and were also analyzed spectroscopically. From the HR-LC-MS trace of the crude product, major lactone **12** had retention time = 2.27 min, and minor lactone **13** had retention time = 2.36 min (reverse phase, eluant acetonitrile/water gradient with 0.1% formic acid). Each isomer exhibited HR-LC-ESI-MS [M + H]+ *m*/*z* calcd for C_36_H_35_N_2_O_4_S_2_+, 623.2033; found, 623.2026.

### 4.3. (3R*,4S*)-2-Isobutyl-2’-((S*,E)-2-isobutyl-1-oxo-3-(thiophen-2-yl)-2,3-dihydroisoquinolin-4(1H)-ylidene)-3-(thiophen-2-yl)-2,3-dihydro-1H-spiro[isoquinoline-4,3’-oxetane]-1,4’-dione (Major Lactone ***12***)

H-1 NMR (400 MHz, chloroform-*d*) δ 8.39 (dd, *J* = 7.7, 1.4 Hz, 1H), 8.10 (dd, *J* = 7.8, 1.3 Hz, 1H), 7.74 (dd, *J* = 7.9, 1.2 Hz, 1H), 7.70 (td, *J* = 7.6, 1.3 Hz, 1H), 7.64 (td, J = 7.6, 1.6 Hz, 1H), 7.59 (td, *J* = 7.7, 1.5 Hz, 1H), 7.47 (td, *J* = 7.6, 1.3 Hz, 1H), 7.33 (dd, *J* = 7.7, 1.1 Hz, 1H), 7.16–7.13 (m, 1H), 7.07 (dd, *J* = 5.1, 1.2 Hz, 1H), 6.93 (d, *J* = 3.5 Hz, 1H), 6.89–6.83 (m, 3H), 5.38 (s, 1H), 4.98 (s, 1H), 3.80 (dd, *J* = 13.7, 9.9 Hz, 1H), 3.49 (dd, *J* = 14.0, 10.3 Hz, 1H), 2.53 (dd, *J* = 13.7, 5.2 Hz, 1H), 2.37 (dd, *J* = 14.0, 5.1 Hz, 1H), 1.67–1.52 (m, 1.8H), 0.59 (app t, *J* = 6.4 Hz, 6H), 0.56 (d, *J* = 6.5 Hz, 3H), 0.46 (d, *J* = 6.6 Hz, 3H); C-13 NMR (101 MHz, chloroform-*d*) δ 165.3, 162.5, 162.1, 147.0, 141.6, 138.8, 133.8, 132.2, 130.7, 130.4, 129.7, 129.2, 129.0, 128.8, 128.5, 128.4, 128.3, 127.8, 127.1, 127.0, 126.6, 126.2, 126.1, 125.5, 110.0, 70.3, 61.7, 55.3, 54.4, 51.9, 27.3, 26.4, 20.3, 19.9, 19.8, 19.7.

### 4.4. (3R*,4S*)-2-Isobutyl-2’-((R*,E)-2-isobutyl-1-oxo-3-(thiophen-2-yl)-2,3-dihydroisoquinolin-4(1H)-ylidene)-3-(thiophen-2-yl)-2,3-dihydro-1H-spiro[isoquinoline-4,3’-oxetane]-1,4’-dione (Assigned to Minor Lactone ***13***)

From the crude NMR spectra of the product mixtures, diagnostic H-3 and carbonyl signals for the minor lactone **13** were observed as follows: H-1 NMR (400 MHz, chloroform-*d*) δ 5.36 (s, 1H) and 5.18 (s, 1H); C-13 NMR (101 MHz, chloroform-*d*) δ 165.8 (*beta*-lactone C=O). 

### 4.5. 6-Isobutyl-5H-thieno[3’,2’:4,5]cyclopenta[1,2-c]isoquinoline-5,10(6H)-dione (Purple Ketone ***21***)

H-1 NMR (500 MHz, chloroform-*d*) δ 8.46 (ddd, *J* = 8.2, 1.2, 0.7 Hz, 1H), 8.30 (ddd, *J* = 8.2, 1.4, 0.7 Hz, 1H), 7.67 (ddd, *J* = 8.3, 7.1, 1.4 Hz, 1H), 7.38 (ddd, *J* = 8.2, 7.1, 0.7 Hz, 1H), 7.37 (d, *J* = 4.8 Hz, 1H), 7.18 (d, *J* = 4.8 Hz, 1H), 4.11 (d, *J* = 7.7 Hz, 2H), 2.28–2.37 (m, 1H), 1.05 (d, *J* = 6.7 Hz, 6H); C-13 NMR (125 MHz, chloroform-*d*, partial, in mixture with **26**) δ 186.1, 163.9, 144.1, 133.9, 131.6, 128.7, 126.0, 125.9, 122.6, 121.8, 111.2, 53.1, 29.0, 20.12; HR-LC-ESI–MS [M + H]^+^ *m*/*z* calcd for C_18_H_16_NO_2_S+, 310.0896; found, 310.0919. 

### 4.6. 2-Isobutyl-4-(thiophen-2-yl)isoquinolin-1(2H)-one (Decarbonylated Product ***26***, Partially Purified)

H-1 NMR: (500 MHz, chloroform-*d*) δ 8.51 (ddd, *J* = 8.2, 1.4, 0.6 Hz, 1H), 7.79 (ddd, *J* = 8.2, 1.2, 0.6 Hz, 1H), 7.65 (ddd, *J* = 8.2, 7.1, 1.5 Hz, 1H), 7.53 (ddd, *J* = 8.2, 7.1, 1.2 Hz, 1H), 7.39 (dd, *J* = 4.8, 1.6 Hz, 1H, presumed thienyl H-5′), 7.17 (s, 1H, presumed H-3), 7.16 (overlapped dd, *J* = 4.6, 1.4 Hz, 1H), 7.15 (overlapped t, *J* = 4.6 Hz, 1H), 3.86 (d, *J* = 7.4 Hz, 2H), 2.30–2.18 (m, 1H), 0.99 (d, *J* = 6.7 Hz, 6H); C-13 NMR (125 MHz, chloroform-*d*, assignments by HSQC) δ 161.8 (C-1, C=O), 137.4 (C-4a/8a), 136.3 (C-4a/8a), 132.6 (C-3), 132.5 (thienyl C-2′), 132.3 (C-6/7), 128.3 (C-8), 127.6 (thienyl C-3′/4′), 127.5 (thienyl C-3′/4′), 127.0 (C-6/7), 125.6 (thienyl C-5′), 124.4 (C-5), 111.6 thienyl C-4), 56.7 (*i*-Bu), 28.4 (*i*-Bu), 20.06 (*i*-Bu); HR-LC-ESI–MS [M + H]^+^ *m*/*z* calcd for C_17_H_18_NOS^+^, 284.1104; found, 284.1130. 

### 4.7. Calculations

Calculations were carried out by using Gaussian 09 or Gaussian 16. Geometry optimizations were carried out at B3LYP/6-31G(d) and were followed by frequency calculations. These confirmed the stationary points as minima and provided also zero-point vibrational energy corrections. Conformational searching was performed manually, and the lowest energy conformation was chosen in each case. Energies reported are the electronic energy plus zero-point energy correction. For the structure used as an analogue of **19a**, initial optimization was carried out while holding the labile C (4°)–CO bond “closed”, with the optimized length found in the analogue of **19b** (1.76 Å) held fixed using the opt = modredundant feature. Further optimization once this constraint was released afforded the ring-opened structure in which the C (4°)–CO bond had cleaved. Computed structures are given in the Supplementary Materials.

## Figures and Tables

**Table 1:** Products from the reaction of cis THIQ carboxylic acid **4** with T3P. NMR yields are based on H-1 NMR analysis with *cis*-stilbene as the internal integration standard.

Product	NMR Yield	Structural Evidence
Allene isomers **9**, **10**, and **11**	58% combined	Previously isolated and characterized; X-ray structure of major isomer **9**
Major lactone **12**	13%	NMR, LC-MS, X-ray structure
Minor lactone **13**	3%	Stereochem tentative; NMR signals in mixture, LC-MS
Purple ketone **21**	6%	X-ray structure, NMR, MS
Decarbonylated DHIQ product **26**	4%	NMR, MS, comparison to structurally related compounds
*Trans* acid **1**	10%	Comparison to the known compound
Recovered **2** (sm)	<1%	Comparison to the known compound

## Data Availability

Not applicable.

## Supplementary Materials

The following supporting information can be downloaded: Scanned ^1^H-NMR spectra for **1**, **9**, and **10**–**13**; **21** and **26**; and **12**; LC-MS-SIM traces for **9**–**11**; **12** and **13**; **21**; and **26**; optimized geometries of enols related to **21**; computed structure(s) of model(s) of **19a** and **19b** and enols related to **20;** and crystal data for **12** (CCDC# 2123878) and **21** (CCDC# 2123879).

## Sample Availability

Samples of the compounds are not available from the authors.
