# Supplementary material for: Stereoelectronic Features of a Complex Ketene Dimerization Reaction
Source: Molecules. 2021 Dec 23;27(1):66. doi: 10.3390/molecules27010066 (PMC8746406; doi:10.3390/molecules27010066)

# Supplementary Materials

# Stereoelectronic features of a complex ketene dimerization reaction

Robert D. Barrows[a], Mark J. Dresel[a], Thomas J. Emge[a], Paul R. Rablen[b], and Spencer Knapp[a,*]

[a] *Department of Chemistry and Chemical Biology, Rutgers – The State University of New Jersey, 123 Bevier Road, Piscataway, NJ 08854, USA*

[b]*Department of Chemistry and Biochemistry, Swarthmore College, 500 College Avenue, Swarthmore, PA 19081, USA*

## Index

| **Scanned Spectra:** | **Page** |
|---|---|
| $^{1}$H NMR crude spectrum for **1**, **9**, and **10**-**13** | SI – 3 |
| $^{1}$H NMR spectrum for **21** and **26** | SI – 4 |
| $^{13}$C NMR spectrum for **21** and **26** | SI – 5 |
| $^{13}$C NMR spectrum for **26** | SI – 6 |
| $^{1}$H -$^{13}$C HSQC spectrum for **26** | SI – 7 |
| $^{1}$H NMR spectrum for **12** | SI – 8 |
| $^{13}$C NMR spectrum for **12** | SI – 9 |
| LC-MS-SIM Trace for **9**-**11** | SI – 10 |
| LC-MS-SIM Trace for **12**, **13** | SI – 11 |
| LC-MS-SIM Trace for **21** | SI – 12 |
| LC-MS-SIM Trace for **26** | SI – 13 |
| Optimized geometries of enols related to **20** | SI – 14-19 |
| Structure(s) of model(s) of **19a** | SI – 20-24 |
| Structure of model of **19b** | SI – 25-26 |
| Tables SI-1. Crystallographic Tables for major lactone **12** | SI – 27-39 |

Tables SI-2. Crystallographic Tables for purple ketone **21** SI – 40-48

Figure SI-1. Fully-labeled ORTEP diagram of major lactone **12** SI – 49

Figure SI-2. Fully-labeled ORTEP diagram of major lactone **21** SI – 50

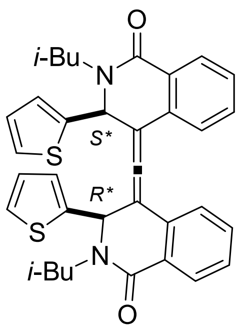

**9**

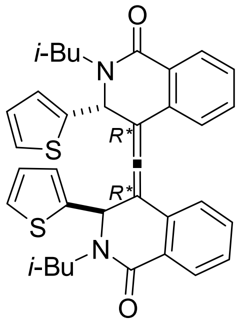

**10** + **11**

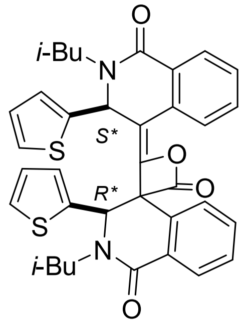

**12**

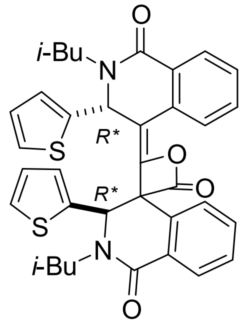

**13**

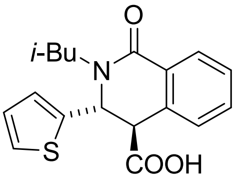

**1**

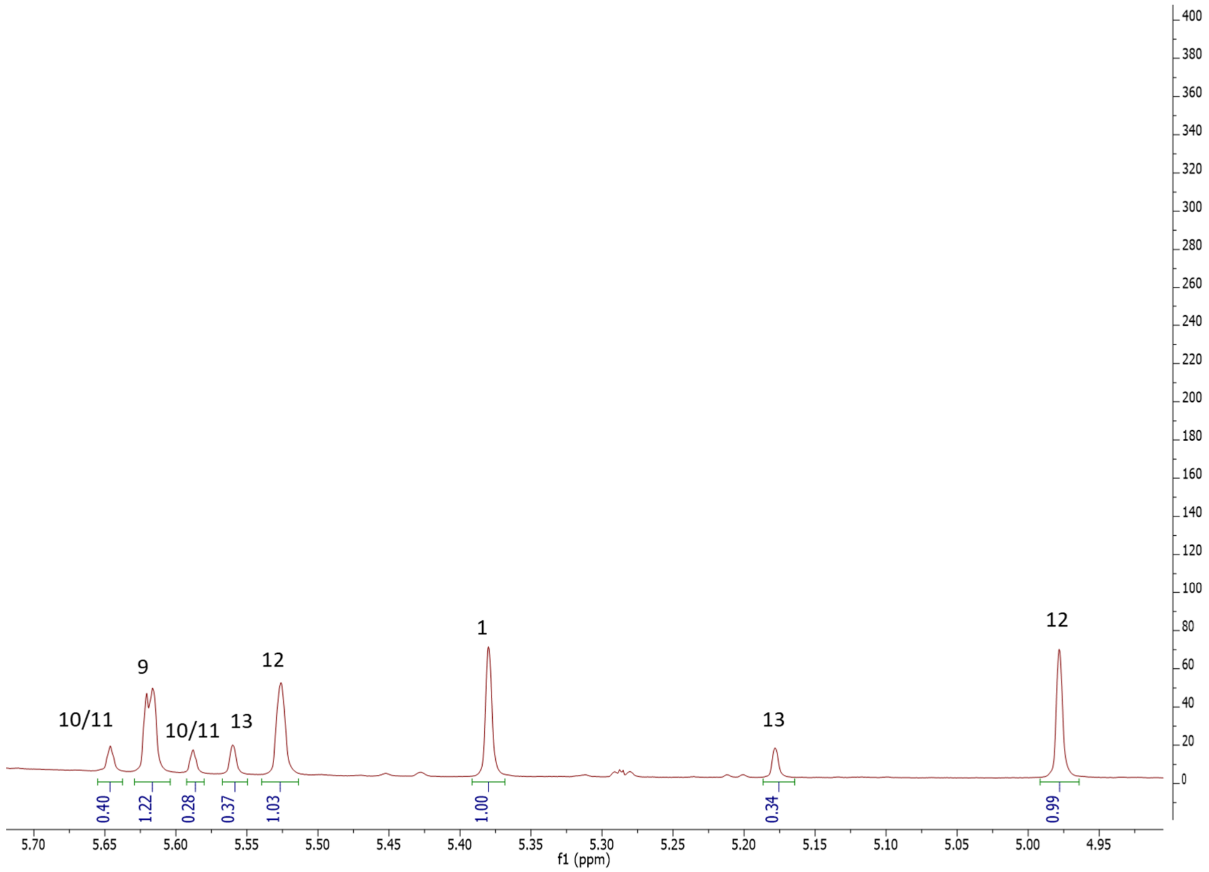

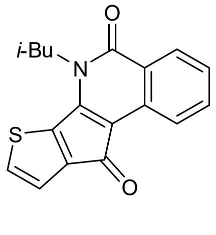
O
i-Bu
N
S
O

**21**

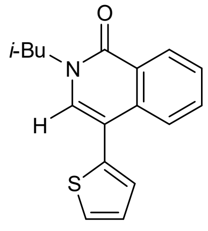
O
i-Bu
N
H
S

**26**

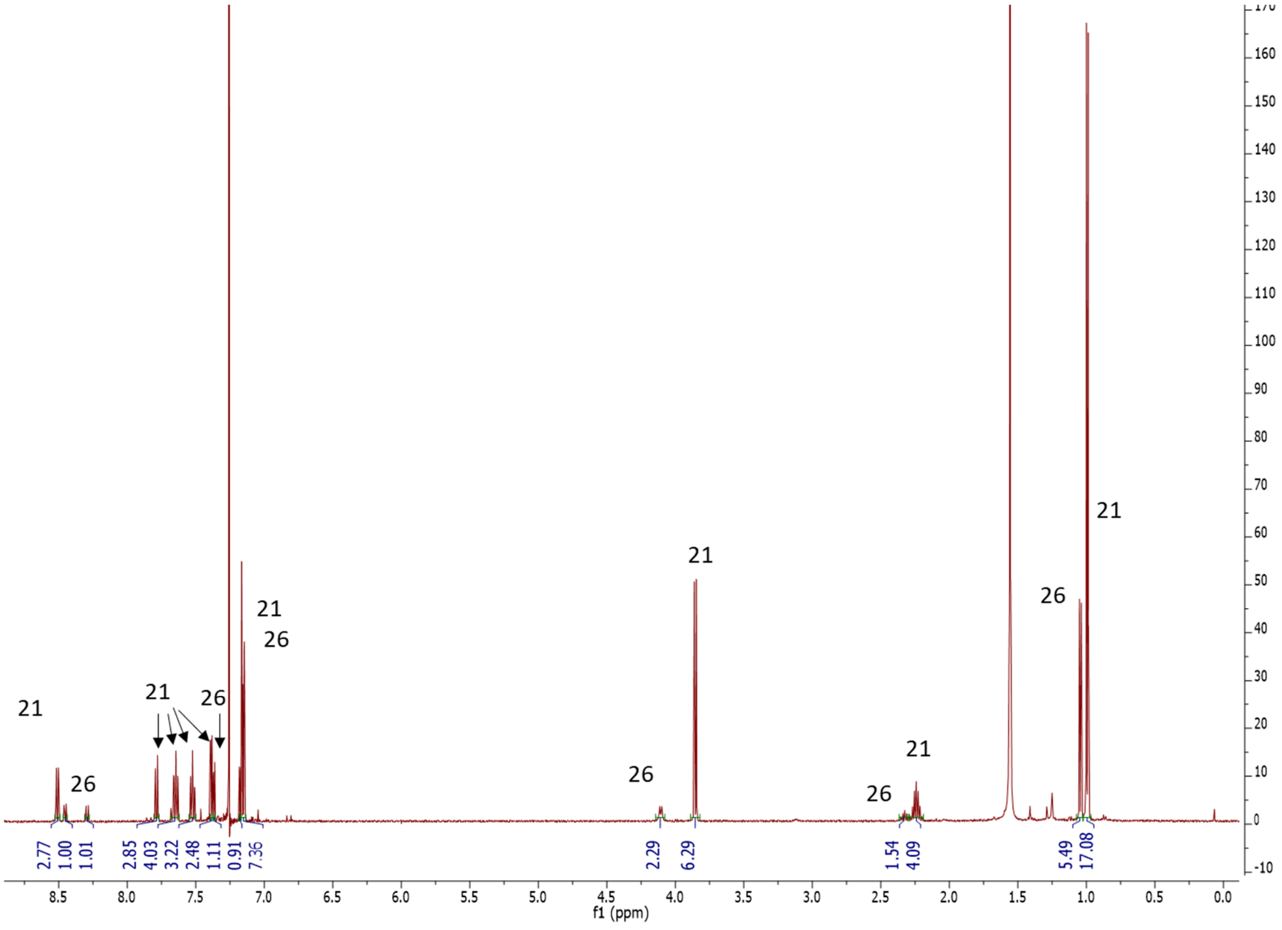
21
26
21
26
21
26
26
21
21
26
21
26
21
2.77
1.00
1.01
2.85
4.03
3.22
2.48
1.11
0.91
7.36
2.29
6.29
1.54
4.09
5.49
17.08
8.5 8.0 7.5 7.0 6.5 6.0 5.5 5.0 4.5 4.0 3.5 3.0 2.5 2.0 1.5 1.0 0.5 0.0
f1 (ppm)
160 150 140 130 120 110 100 90 80 70 60 50 40 30 20 10 0 -10

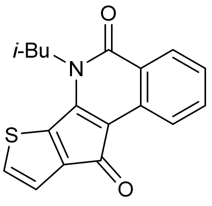
O
i-Bu
N
S
O

**21**

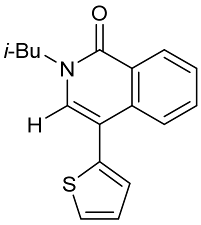
O
i-Bu
N
H
S

**26**

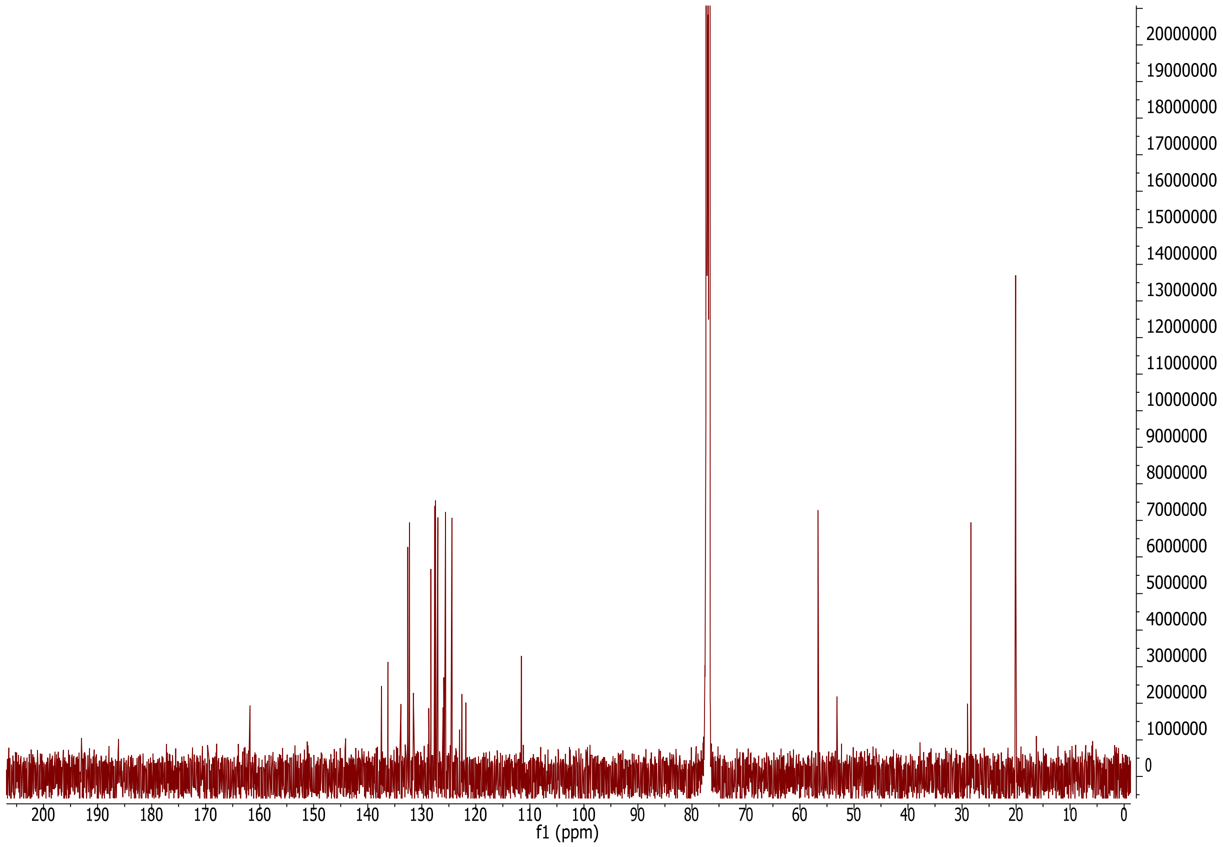
20000000
19000000
18000000
17000000
16000000
15000000
14000000
13000000
12000000
11000000
10000000
9000000
8000000
7000000
6000000
5000000
4000000
3000000
2000000
1000000
0
200 190 180 170 160 150 140 130 120 110 100 90 80 70 60 50 40 30 20 10 0
f1 (ppm)

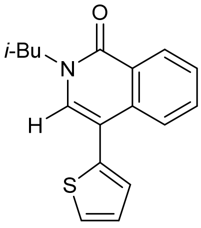
O
i-Bu
N
H
S

**26**

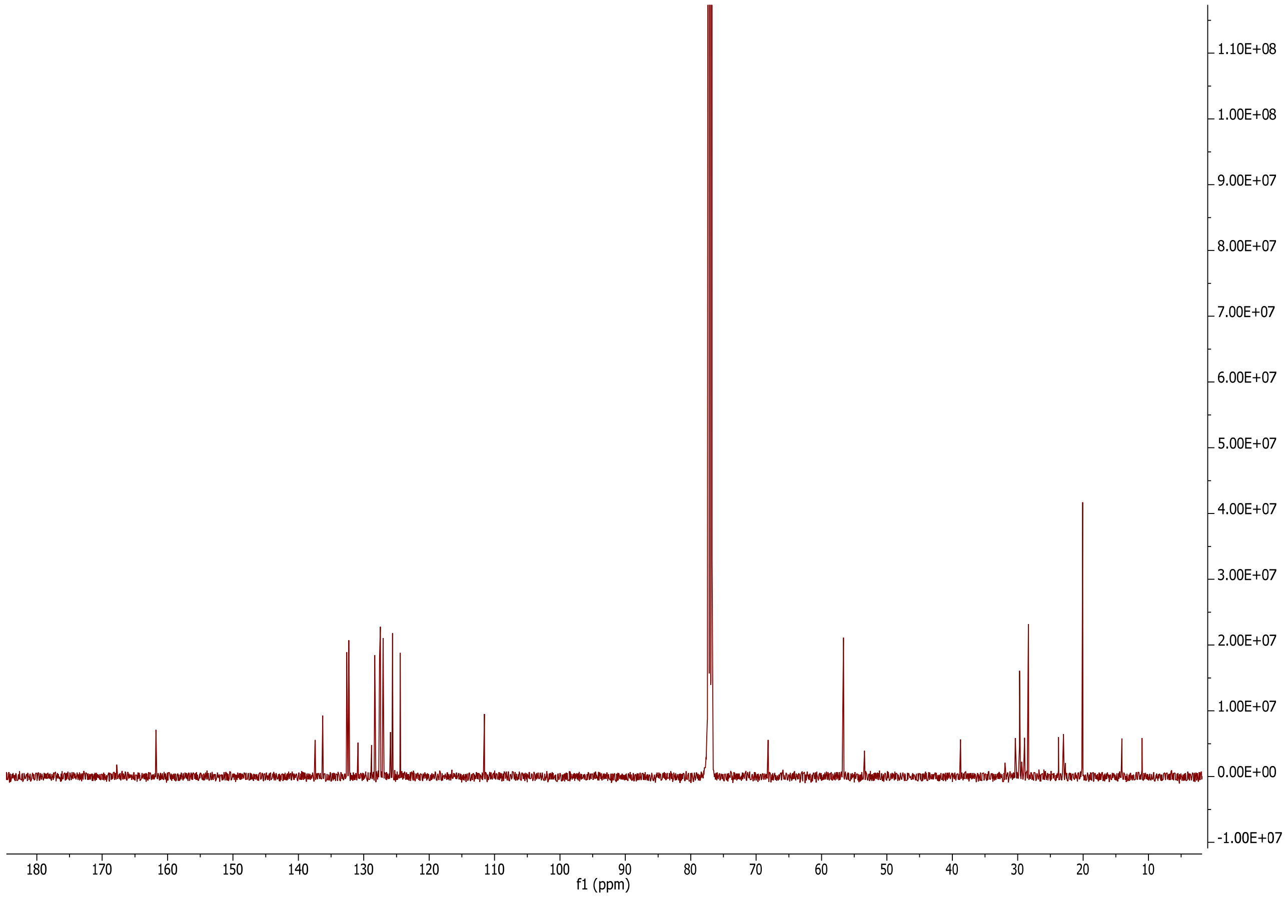
1.10E+08
1.00E+08
9.00E+07
8.00E+07
7.00E+07
6.00E+07
5.00E+07
4.00E+07
3.00E+07
2.00E+07
1.00E+07
0.00E+00
-1.00E+07
180
170
160
150
140
130
120
110
100
90
80
70
60
50
40
30
20
10
f1 (ppm)

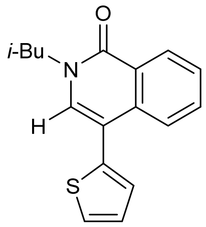
O
i-Bu
N
H
S

**26**

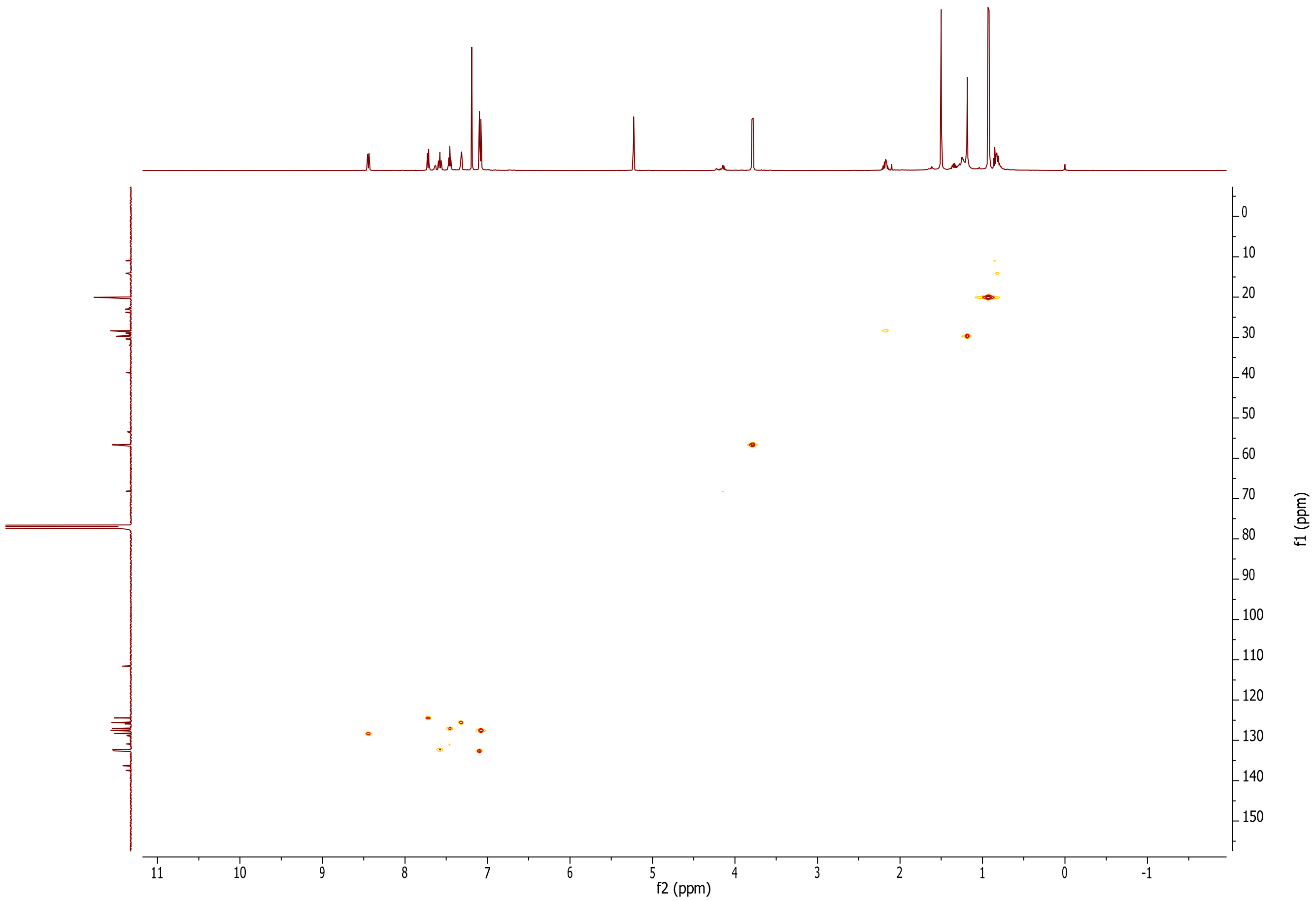
f2 (ppm)
f1 (ppm)
11
10
9
8
7
6
5
4
3
2
1
0
-1
0
10
20
30
40
50
60
70
80
90
100
110
120
130
140
150

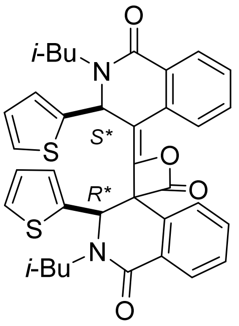


**12**

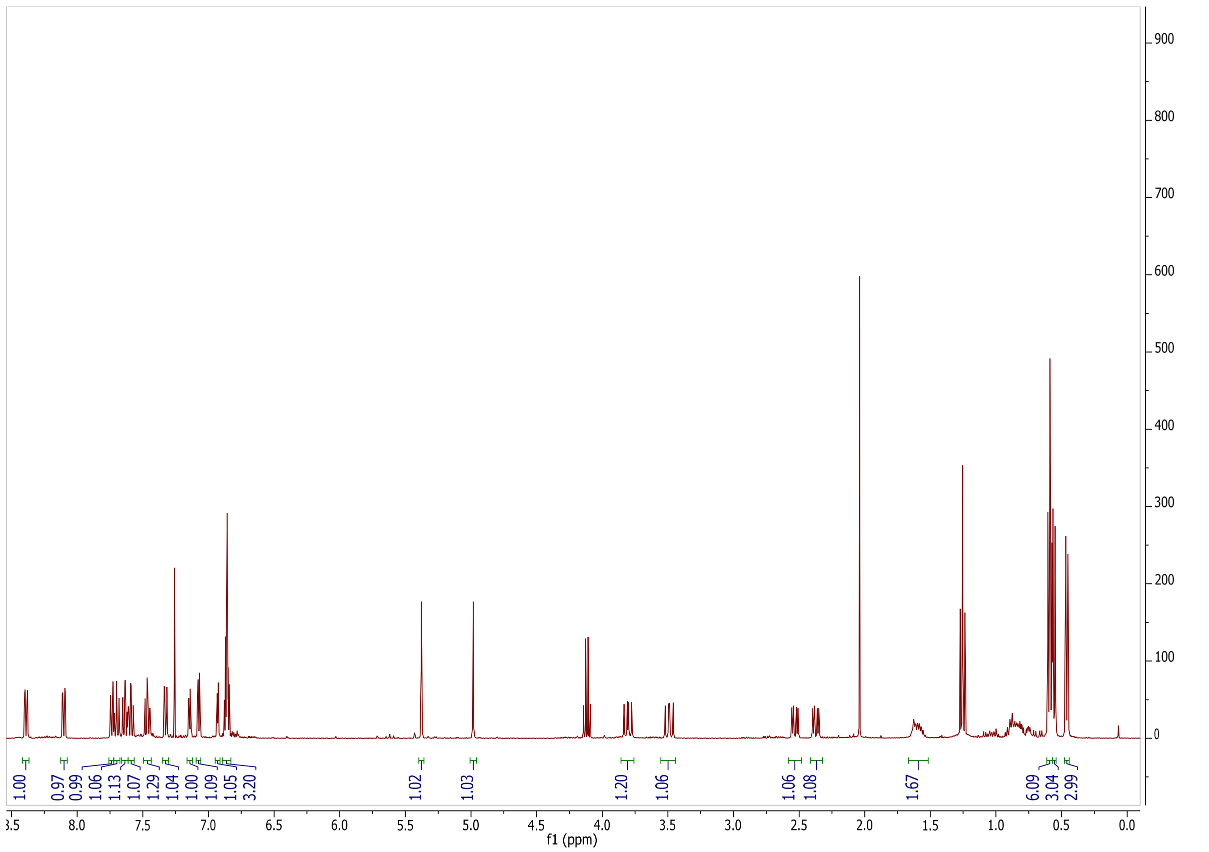

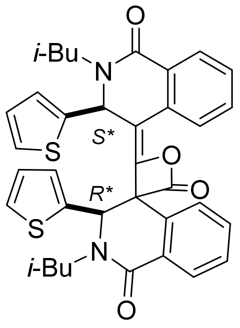


**12**

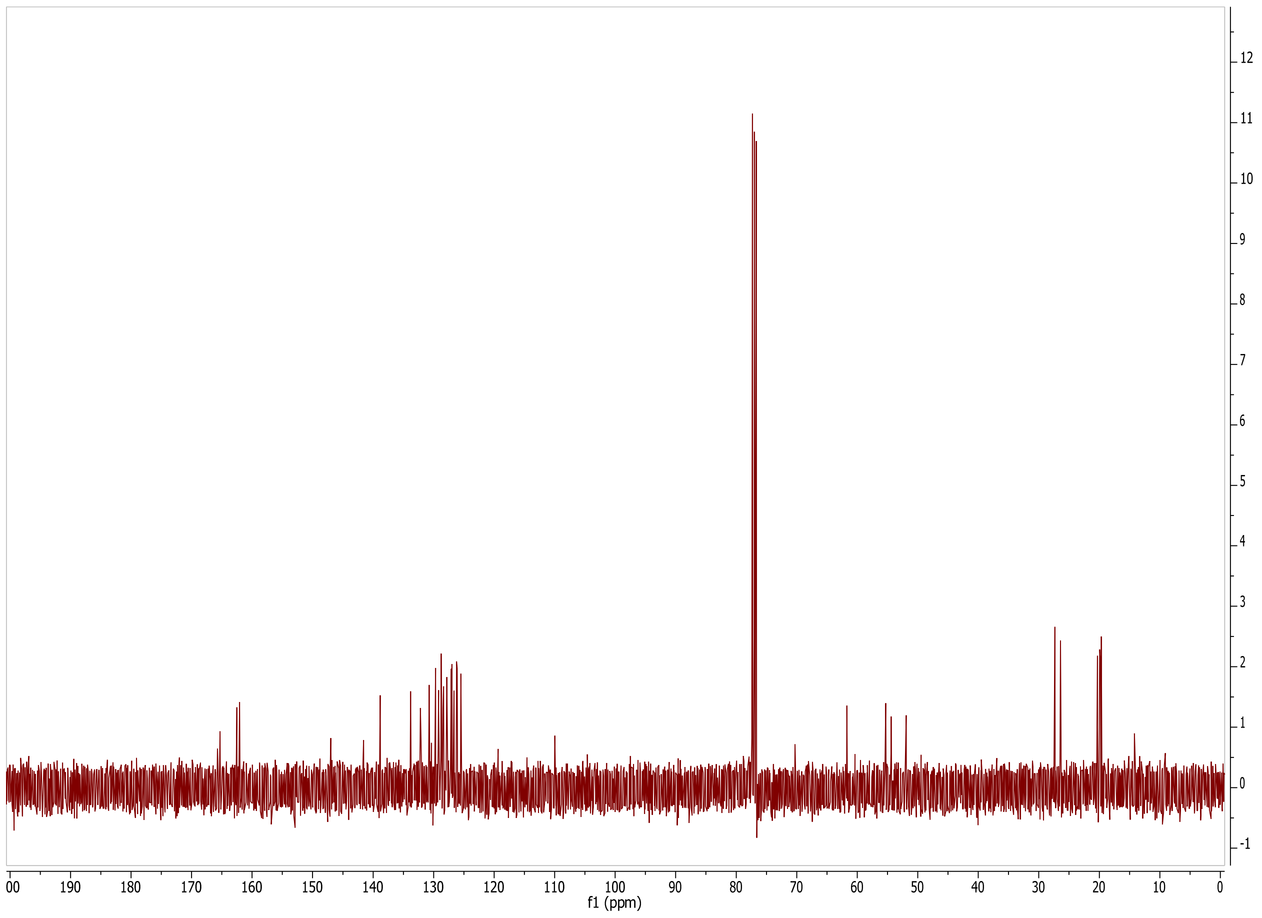

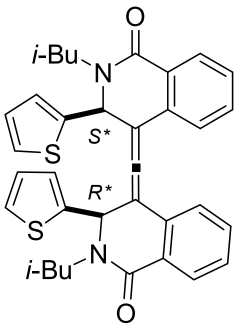
O
i-Bu
N
S*
S
R*
S
N
i-Bu
O

**9**

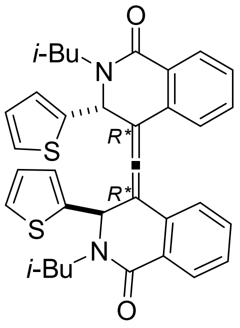
O
i-Bu
N
R*
S
R*
S
N
i-Bu
O

**10** + **11**

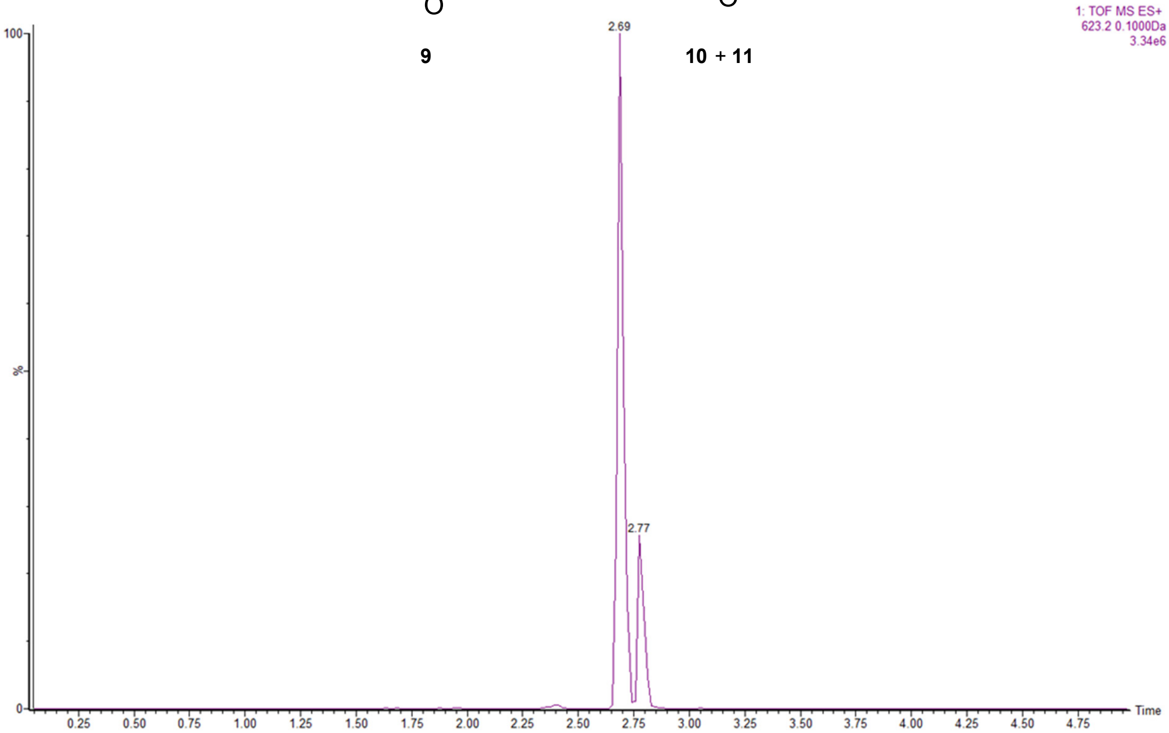
1: TOF MS ES+
623.2 0.1000Da
3.34e6
100
%
0
2.69
2.77
0.25
0.50
0.75
1.00
1.25
1.50
1.75
2.00
2.25
2.50
2.75
3.00
3.25
3.50
3.75
4.00
4.25
4.50
4.75
Time

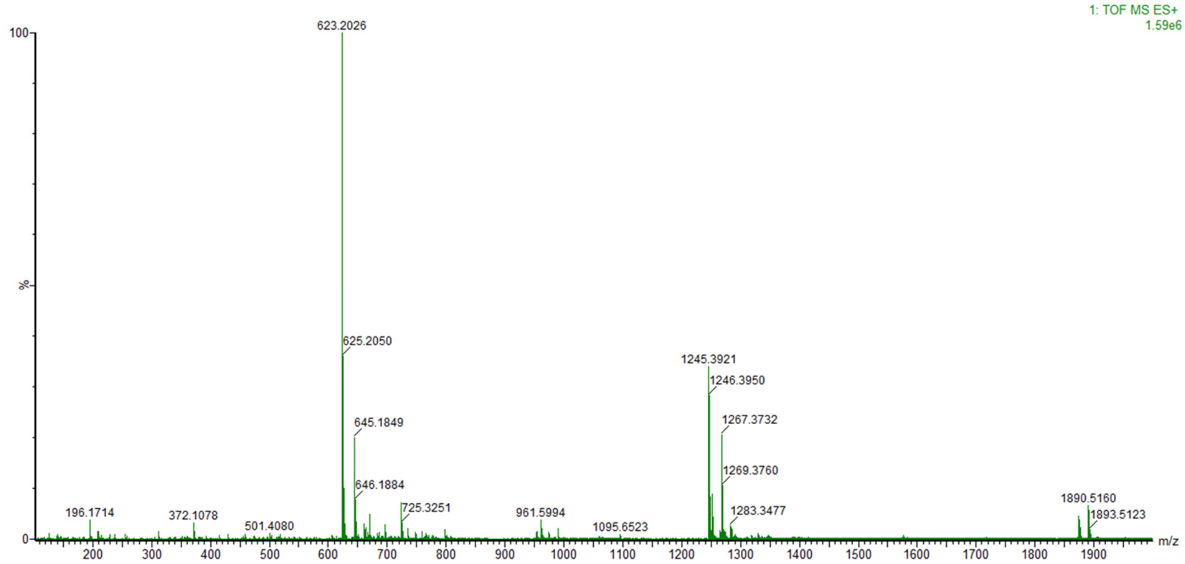
1: TOF MS ES+
1.59e6
100
%
0
623.2026
625.2050
645.1849
646.1884
725.3251
196.1714
372.1078
501.4080
961.5994
1095.6523
1245.3921
1246.3950
1267.3732
1269.3760
1283.3477
1890.5160
1893.5123
200
300
400
500
600
700
800
900
1000
1100
1200
1300
1400
1500
1600
1700
1800
1900
m/z

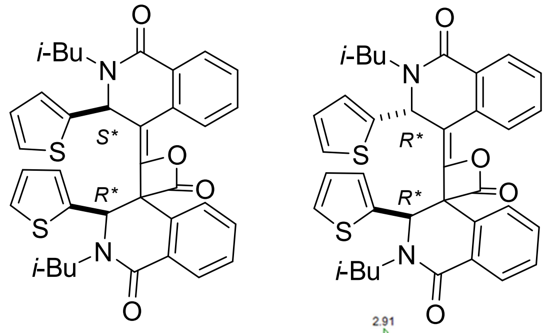
i-Bu
N
S
S*
O
O
O
R*
S
N
i-Bu
O
i-Bu
N
S
R*
O
O
O
R*
S
N
i-Bu
O

12 13

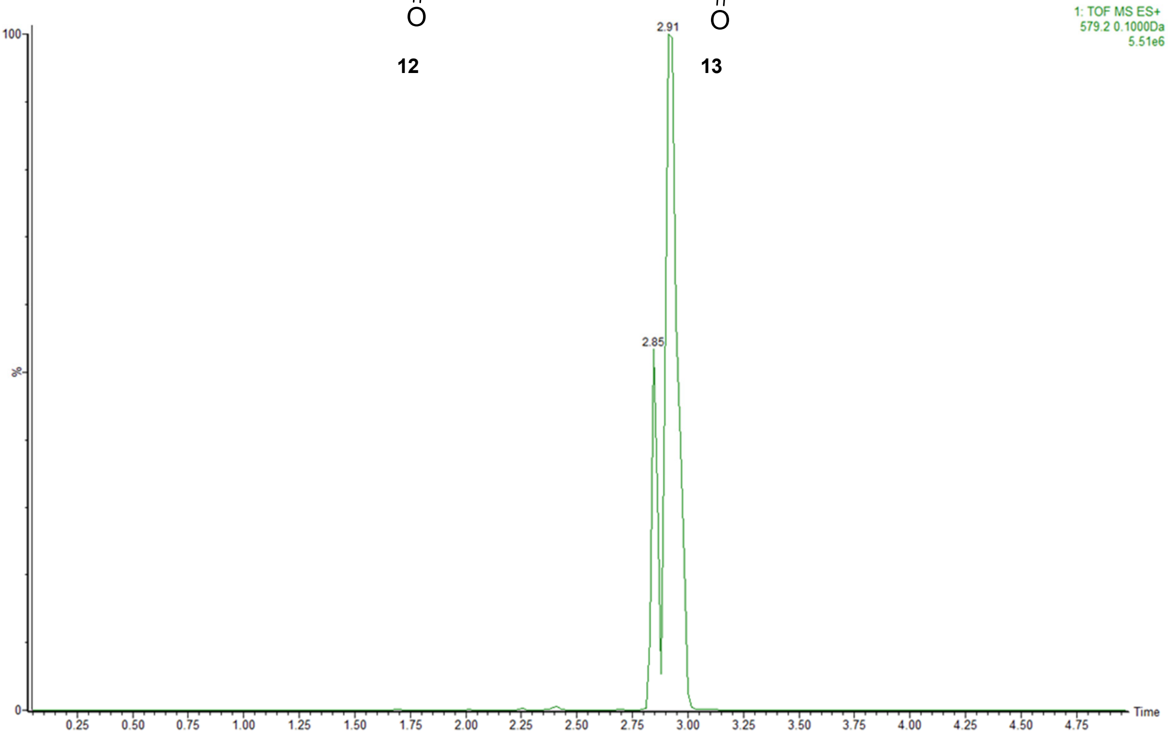
1: TOF MS ES+
579.2 0.1000Da
5.51e6
2.91
2.85
100
%
0
Time
0.25 0.50 0.75 1.00 1.25 1.50 1.75 2.00 2.25 2.50 2.75 3.00 3.25 3.50 3.75 4.00 4.25 4.50 4.75

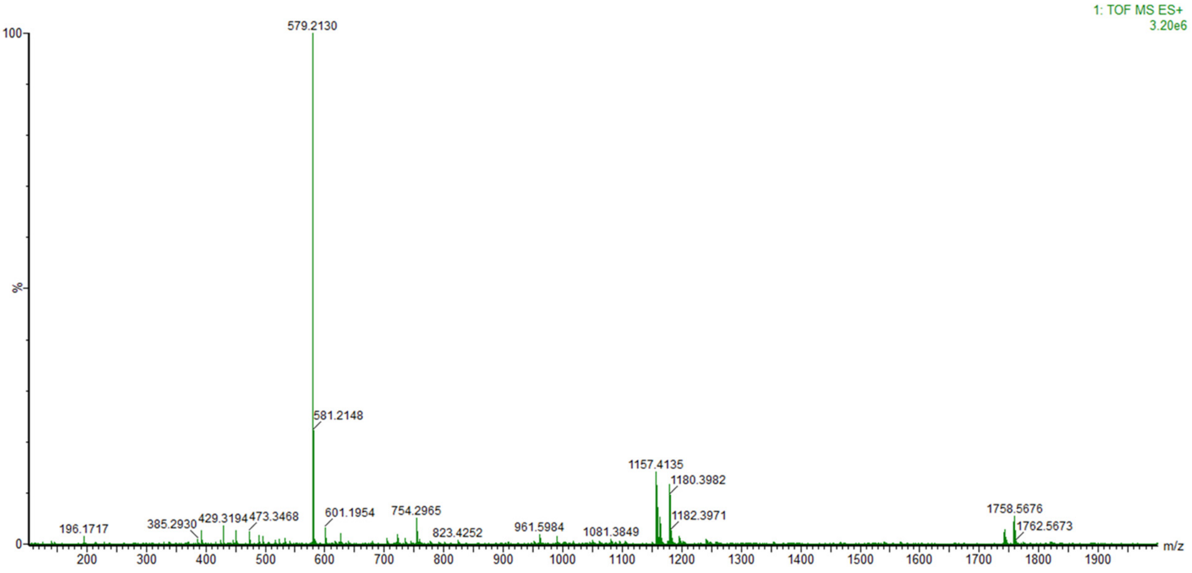
1: TOF MS ES+
3.20e6
579.2130
581.2148
1157.4135
1180.3982
1182.3971
1758.5676
1762.5673
196.1717
385.2930
429.3194
473.3468
601.1954
754.2965
823.4252
961.5984
1081.3849
100
%
0
m/z
200 300 400 500 600 700 800 900 1000 1100 1200 1300 1400 1500 1600 1700 1800 1900

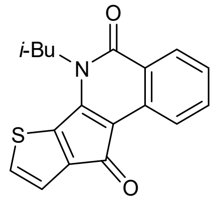

**21**

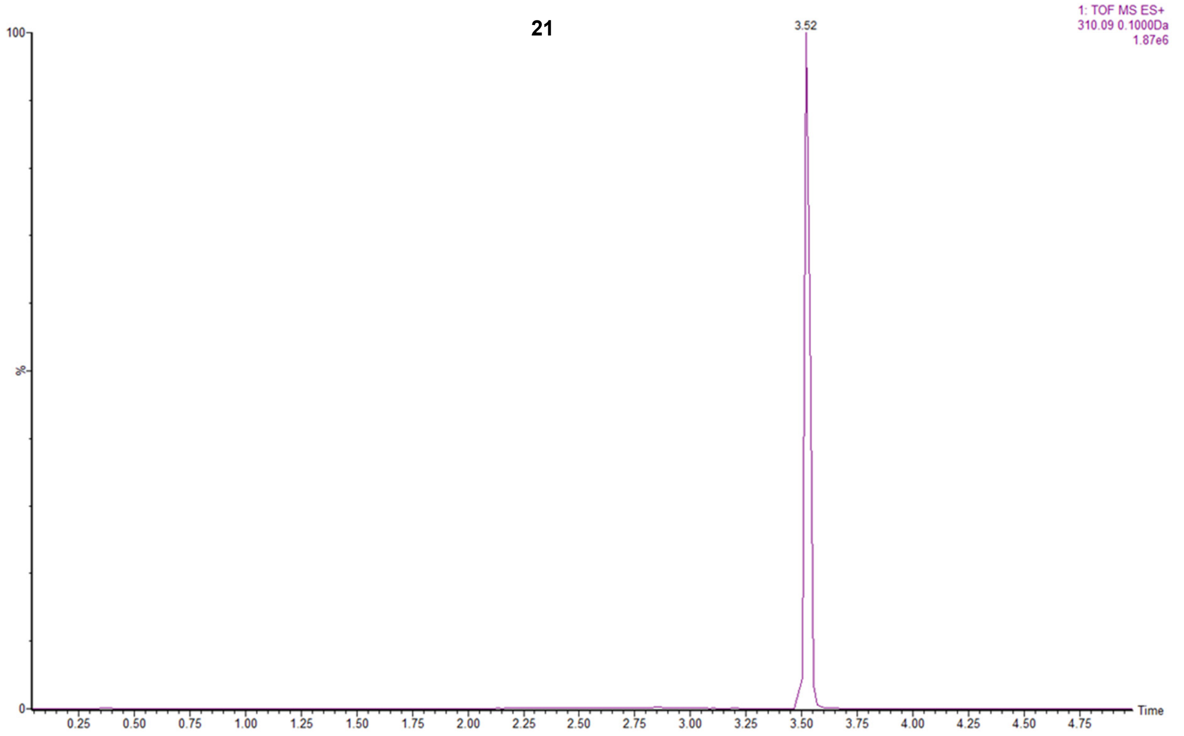

3.52
1: TOF MS ES+
310.09 0.1000Da
1.87e6
100
%
0
Time
0.25 0.50 0.75 1.00 1.25 1.50 1.75 2.00 2.25 2.50 2.75 3.00 3.25 3.50 3.75 4.00 4.25 4.50 4.75

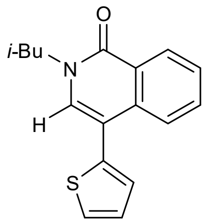
i-Bu
N
O
H
S

**26**

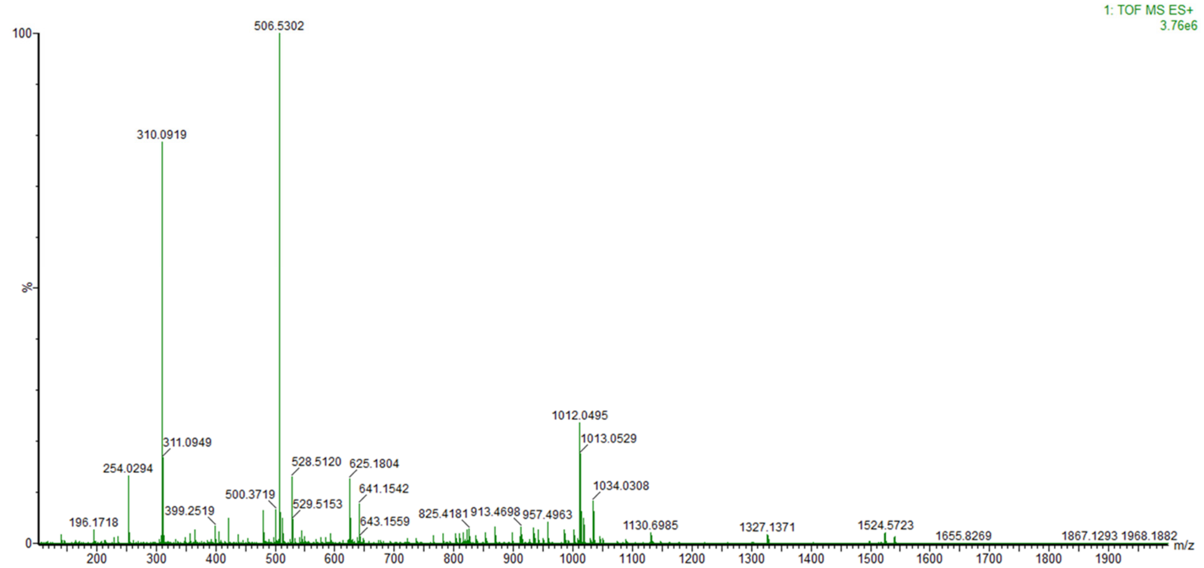
1: TOF MS ES+
3.76e6
506.5302
310.0919
1012.0495
1013.0529
311.0949
254.0294
528.5120
625.1804
641.1542
1034.0308
500.3719
529.5153
399.2519
196.1718
643.1559
825.4181
913.4698
957.4963
1130.6985
1327.1371
1524.5723
1655.8269
1867.1293
1968.1882
m/z
%

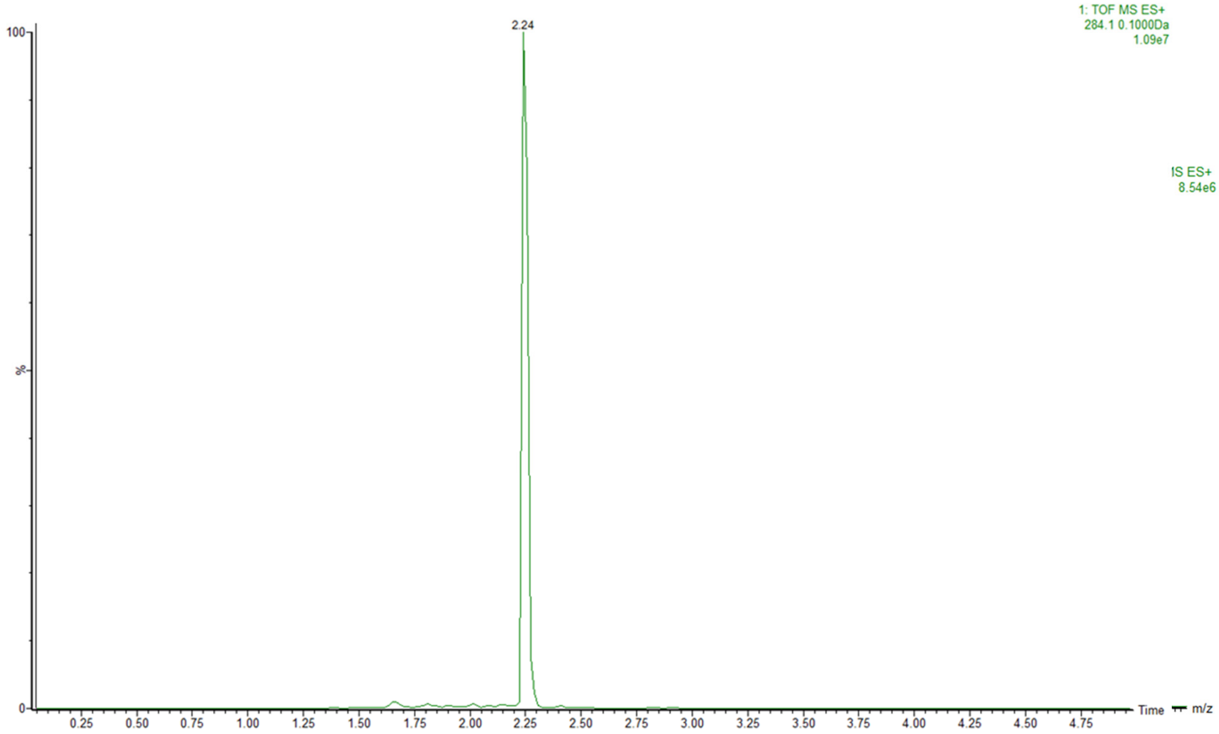
1: TOF MS ES+
284.1 0.1000Da
1.09e7
IS ES+
8.54e6
2.24
Time
m/z
%

B3LYP/6-31G(d) fopt freq

----------------------------------------------------------------------

Protonated enolate (red H) generated from ketene **15** by internal C-3' thiophene attack, H-3' trans to H-3

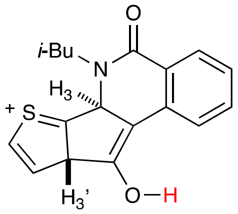


----------------------------------------------------------------------

Input orientation:

| Center Number | Atomic Number | Atomic Type | Coordinates (Angstroms) X | Y | Z |
|---|---|---|---|---|---|
| 1 | 6 | 0 | -0.812837 | -0.996318 | 0.044073 |
| 2 | 6 | 0 | 0.273303 | 0.054643 | 0.244477 |
| 3 | 7 | 0 | -0.054081 | 1.251315 | -0.525813 |
| 4 | 6 | 0 | -1.377971 | 1.710022 | -0.478233 |
| 5 | 6 | 0 | -2.422133 | 0.791358 | 0.098965 |
| 6 | 6 | 0 | -3.702878 | 1.303440 | 0.314998 |
| 7 | 6 | 0 | -4.710749 | 0.478530 | 0.816377 |
| 8 | 6 | 0 | -4.448675 | -0.865418 | 1.104670 |
| 9 | 6 | 0 | -3.175576 | -1.392547 | 0.892129 |
| 10 | 6 | 0 | -2.158953 | -0.570313 | 0.385124 |
| 11 | 6 | 0 | -0.384104 | -2.036526 | -0.714886 |
| 12 | 8 | 0 | -1.030971 | -3.108932 | -1.177028 |
| 13 | 8 | 0 | -1.672338 | 2.821138 | -0.889486 |
| 14 | 6 | 0 | 1.429443 | -0.785447 | -0.228253 |
| 15 | 16 | 0 | 2.871613 | -1.083820 | 0.551394 |
| 16 | 6 | 0 | 3.100582 | -2.648780 | -0.289880 |
| 17 | 6 | 0 | 2.077079 | -2.941843 | -1.102770 |
| 18 | 6 | 0 | 1.051755 | -1.850132 | -1.204970 |
| 19 | 6 | 0 | 1.019268 | 2.229635 | -0.776204 |
| 20 | 6 | 0 | 1.779729 | 2.004736 | -2.095307 |
| 21 | 6 | 0 | 2.944031 | 3.001687 | -2.190857 |
| 22 | 6 | 0 | 0.850648 | 2.099083 | -3.312141 |
| 23 | 1 | 0 | 0.413453 | 0.311800 | 1.307861 |
| 24 | 1 | 0 | -3.892067 | 2.344522 | 0.076463 |
| 25 | 1 | 0 | -5.704075 | 0.883017 | 0.984063 |
| 26 | 1 | 0 | -5.234603 | -1.498491 | 1.505095 |
| 27 | 1 | 0 | -2.961710 | -2.424133 | 1.163757 |
| 28 | 1 | 0 | 3.996124 | -3.218786 | -0.074970 |
| 29 | 1 | 0 | 2.025188 | -3.840837 | -1.707391 |
| 30 | 1 | 0 | 1.024259 | -1.484305 | -2.248143 |
| 31 | 1 | 0 | 1.716212 | 2.199532 | 0.075176 |
| 32 | 1 | 0 | 0.549007 | 3.215422 | -0.783534 |
| 33 | 1 | 0 | 2.213388 | 0.987882 | -2.071983 |
| 34 | 1 | 0 | 3.521329 | 2.839662 | -3.106692 |

| | | | | | |
|---|---|---|---|---|---|
| 35 | 1 | 0 | 2.570858 | 4.032478 | -2.212110 |
| 36 | 1 | 0 | 3.630364 | 2.912194 | -1.340133 |
| 37 | 1 | 0 | 0.032048 | 1.373140 | -3.255431 |
| 38 | 1 | 0 | 0.400205 | 3.095639 | -3.380303 |
| 39 | 1 | 0 | 1.405305 | 1.915309 | -4.238486 |
| 40 | 1 | 0 | -1.952978 | -3.116503 | -0.860123 |

---------------------------------------------------------------------

Stoichiometry    C18H18NO2S(1+)
Framework group  C1[X(C18H18NO2S)]
Deg. of freedom   114
Full point group                 C1      NOp   1
Largest Abelian subgroup         C1      NOp   1
Largest concise Abelian subgroup C1      NOp   1

Zero-point correction=                           0.323732 (Hartree/Particle)
Thermal correction to Energy=                    0.343030
Thermal correction to Enthalpy=                  0.343975
Thermal correction to Gibbs Free Energy=         0.275894
Sum of electronic and zero-point Energies=          -1299.522321
Sum of electronic and thermal Energies=             -1299.503023
Sum of electronic and thermal Enthalpies=           -1299.502079
Sum of electronic and thermal Free Energies=        -1299.570159

B3LYP/6-31G(d) fopt freq

---

Protonated enolate (red H) generated from ketene **15** by internal C-3' thiophene attack, H-3' cis to H-3

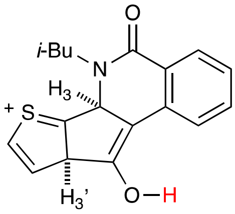


---

Input orientation:

| Center Number | Atomic Number | Atomic Type | Coordinates (Angstroms) X | Y | Z |
|---|---|---|---|---|---|
| 1 | 6 | 0 | -0.582456 | -1.074065 | 0.442822 |
| 2 | 6 | 0 | 0.620593 | -0.121748 | 0.367454 |
| 3 | 7 | 0 | 0.381893 | 1.069494 | -0.410248 |
| 4 | 6 | 0 | -0.884895 | 1.498755 | -0.793994 |
| 5 | 6 | 0 | -2.067140 | 0.685833 | -0.363337 |
| 6 | 6 | 0 | -3.340024 | 1.198052 | -0.620775 |
| 7 | 6 | 0 | -4.475769 | 0.497932 | -0.215033 |
| 8 | 6 | 0 | -4.344595 | -0.717078 | 0.463476 |
| 9 | 6 | 0 | -3.080969 | -1.249413 | 0.712638 |
| 10 | 6 | 0 | -1.929449 | -0.556734 | 0.291165 |
| 11 | 6 | 0 | -0.163191 | -2.378491 | 0.462203 |
| 12 | 8 | 0 | -0.880557 | -3.500120 | 0.412177 |
| 13 | 8 | 0 | -1.021501 | 2.533817 | -1.428845 |
| 14 | 6 | 0 | 1.491159 | -1.175559 | -0.275716 |
| 15 | 16 | 0 | 2.207312 | -1.234787 | -1.787429 |
| 16 | 6 | 0 | 2.405541 | -3.016501 | -1.688239 |
| 17 | 6 | 0 | 1.912730 | -3.535576 | -0.558729 |
| 18 | 6 | 0 | 1.361508 | -2.512703 | 0.393301 |
| 19 | 6 | 0 | 1.503991 | 2.020971 | -0.591741 |
| 20 | 6 | 0 | 1.500305 | 3.243391 | 0.347489 |
| 21 | 6 | 0 | 2.682272 | 4.152004 | -0.022207 |
| 22 | 6 | 0 | 1.537458 | 2.857572 | 1.832359 |
| 23 | 1 | 0 | 1.012291 | 0.134396 | 1.363615 |
| 24 | 1 | 0 | -3.415742 | 2.148951 | -1.136907 |
| 25 | 1 | 0 | -5.462061 | 0.905029 | -0.414114 |
| 26 | 1 | 0 | -5.226797 | -1.246658 | 0.809873 |
| 27 | 1 | 0 | -2.994892 | -2.154969 | 1.308355 |
| 28 | 1 | 0 | 2.877190 | -3.520500 | -2.523022 |
| 29 | 1 | 0 | 1.937862 | -4.592987 | -0.320182 |
| 30 | 1 | 0 | 1.830340 | -2.573261 | 1.388035 |
| 31 | 1 | 0 | 1.469166 | 2.361360 | -1.629722 |
| 32 | 1 | 0 | 2.434887 | 1.455427 | -0.453685 |
| 33 | 1 | 0 | 0.572672 | 3.792903 | 0.152060 |
| 34 | 1 | 0 | 2.673816 | 5.060017 | 0.589091 |
| 35 | 1 | 0 | 3.643355 | 3.648814 | 0.147629 |
| 36 | 1 | 0 | 2.641155 | 4.458987 | -1.073247 |
| 37 | 1 | 0 | 0.657010 | 2.274919 | 2.130540 |
| 38 | 1 | 0 | 2.438827 | 2.277969 | 2.075646 |
| 39 | 1 | 0 | 1.550761 | 3.754232 | 2.460590 |
| 40 | 1 | 0 | -1.799751 | -3.305927 | 0.142768 |

---

Stoichiometry C18H18NO2S(1+)
Framework group C1[X(C18H18NO2S)]
Deg. of freedom 114
Full point group C1 NOp 1
Largest Abelian subgroup C1 NOp 1
Largest concise Abelian subgroup C1 NOp 1

Zero-point correction= 0.324363 (Hartree/Particle)
Thermal correction to Energy= 0.343524
Thermal correction to Enthalpy= 0.344468
Thermal correction to Gibbs Free Energy= 0.276714

Sum of electronic and zero-point Energies= -1299.528035
Sum of electronic and thermal Energies= -1299.508875
Sum of electronic and thermal Enthalpies= -1299.507931
Sum of electronic and thermal Free Energies= -1299.575685

B3LYP/6-31G(d) fopt freq

---

Protonated enolate (red H) generated from ketene **15** by internal thiophene attack by S

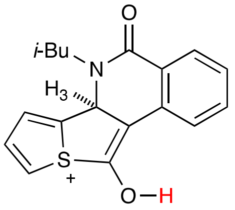


---

Input orientation:

| Center Number | Atomic Number | Atomic Type | X | Y | Z |
|---|---|---|---|---|---|
| 1 | 6 | 0 | -1.091257 | 0.441290 | 0.408964 |
| 2 | 6 | 0 | 0.006132 | -0.627979 | 0.455221 |
| 3 | 7 | 0 | 1.166719 | -0.298751 | -0.347299 |
| 4 | 6 | 0 | 1.430842 | 0.981198 | -0.819252 |
| 5 | 6 | 0 | 0.497847 | 2.085099 | -0.423746 |
| 6 | 6 | 0 | 0.877929 | 3.397453 | -0.714485 |
| 7 | 6 | 0 | 0.072304 | 4.465177 | -0.321903 |
| 8 | 6 | 0 | -1.116827 | 4.228469 | 0.374337 |
| 9 | 6 | 0 | -1.517374 | 2.923525 | 0.655830 |
| 10 | 6 | 0 | -0.716035 | 1.842130 | 0.249860 |
| 11 | 6 | 0 | -2.358618 | -0.032651 | 0.429395 |
| 12 | 8 | 0 | -3.560915 | 0.552645 | 0.386369 |
| 13 | 8 | 0 | 2.431900 | 1.203294 | -1.485067 |
| 14 | 6 | 0 | -0.751009 | -1.843875 | -0.053279 |
| 15 | 16 | 0 | -2.409272 | -1.841652 | 0.679881 |
| 16 | 6 | 0 | -3.081229 | -2.744839 | -0.705947 |
| 17 | 6 | 0 | -2.064001 | -3.161980 | -1.490423 |
| 18 | 6 | 0 | -0.755088 | -2.600060 | -1.164565 |
| 19 | 6 | 0 | 2.273151 | -1.283441 | -0.416712 |
| 20 | 6 | 0 | 3.479065 | -0.997297 | 0.501100 |
| 21 | 6 | 0 | 4.542928 | -2.079429 | 0.263053 |
| 22 | 6 | 0 | 3.092523 | -0.912776 | 1.983928 |
| 23 | 1 | 0 | 0.309527 | -0.792010 | 1.501607 |
| 24 | 1 | 0 | 1.811763 | 3.555987 | -1.242938 |
| 25 | 1 | 0 | 0.376291 | 5.482778 | -0.546428 |
| 26 | 1 | 0 | -1.729906 | 5.061116 | 0.705370 |
| 27 | 1 | 0 | -2.416999 | 2.753238 | 1.241280 |
| 28 | 1 | 0 | -4.117591 | -3.054296 | -0.685486 |
| 29 | 1 | 0 | -2.215931 | -3.839307 | -2.325485 |
| 30 | 1 | 0 | 0.103603 | -2.698383 | -1.818828 |
| 31 | 1 | 0 | 2.610006 | -1.315328 | -1.456465 |
| 32 | 1 | 0 | 1.852623 | -2.263994 | -0.166071 |
| 33 | 1 | 0 | 3.897928 | -0.033075 | 0.193240 |
| 34 | 1 | 0 | 5.438543 | -1.875380 | 0.858453 |
| 35 | 1 | 0 | 4.174796 | -3.073256 | 0.550327 |
| 36 | 1 | 0 | 4.846708 | -2.121233 | -0.788983 |
| 37 | 1 | 0 | 2.389776 | -0.093808 | 2.181433 |
| 38 | 1 | 0 | 2.642834 | -1.851128 | 2.337179 |
| 39 | 1 | 0 | 3.977538 | -0.725744 | 2.600948 |
| 40 | 1 | 0 | -3.493876 | 1.406939 | -0.083594 |

Coordinates (Angstroms)

Stoichiometry C18H18NO2S(1+)
Framework group C1[X(C18H18NO2S)]
Deg. of freedom 114
Full point group C1 NOp 1
Largest Abelian subgroup C1 NOp 1
Largest concise Abelian subgroup C1 NOp 1

Zero-point correction= 0.323193 (Hartree/Particle)
Thermal correction to Energy= 0.342792
Thermal correction to Enthalpy= 0.343736
Thermal correction to Gibbs Free Energy= 0.275579

| | |
|---|---|
| Sum of electronic and zero-point Energies= | -1299.501535 |
| Sum of electronic and thermal Energies= | -1299.481937 |
| Sum of electronic and thermal Enthalpies= | -1299.480992 |
| Sum of electronic and thermal Free Energies= | -1299.549149 |

------------------------------------------------------------------------

Simplified model of lactone structure **19a**: N-isobutyl changed to N-methyl, O changed to CH2, NEt3 changed to tBu, alkene truncated (changes and scissile bond indicated in red)

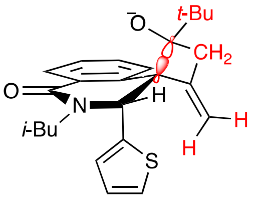


------------------------------------------------------------------------

Geometry optimized with labile C(4°)-C=O bond distance held fixed at 1.76285 Angstroms using opt=modredundant

------------------------------------------------------------------------

Input orientation:

| Center Number | Atomic Number | Atomic Type | Coordinates (Angstroms) X | Y | Z |
|---|---|---|---|---|---|
| 1 | 6 | 0 | -0.704953 | -1.208781 | 1.678309 |
| 2 | 8 | 0 | -0.988214 | -1.981654 | 2.598935 |
| 3 | 7 | 0 | 0.249634 | -0.223552 | 1.826540 |
| 4 | 6 | 0 | 0.676905 | 0.663574 | 0.728960 |
| 5 | 6 | 0 | -0.514426 | 0.946301 | -0.191330 |
| 6 | 6 | 0 | -1.120660 | -0.324914 | -0.649183 |
| 7 | 6 | 0 | -1.296982 | -1.338774 | 0.318203 |
| 8 | 6 | 0 | -1.970409 | -2.522884 | 0.002022 |
| 9 | 6 | 0 | -2.472776 | -2.728127 | -1.279040 |
| 10 | 6 | 0 | -2.289255 | -1.737674 | -2.251804 |
| 11 | 6 | 0 | -1.625867 | -0.553991 | -1.939659 |
| 12 | 6 | 0 | -0.439880 | 2.064778 | -1.203188 |
| 13 | 6 | 0 | 0.565411 | 2.555264 | -1.934145 |
| 14 | 6 | 0 | -1.823152 | 1.886245 | 0.523780 |
| 15 | 8 | 0 | -2.852048 | 1.247581 | 0.930581 |
| 16 | 6 | 0 | -1.812847 | 2.614068 | -0.907086 |
| 17 | 6 | 0 | -1.201102 | 2.856116 | 1.576824 |
| 18 | 6 | 0 | 0.977791 | -0.125190 | 3.075346 |
| 19 | 6 | 0 | 1.958797 | 0.162250 | 0.073851 |
| 20 | 16 | 0 | 2.069653 | -1.337582 | -0.827010 |
| 21 | 6 | 0 | 3.759205 | -1.089565 | -1.149265 |
| 22 | 6 | 0 | 4.209484 | 0.080783 | -0.604783 |
| 23 | 6 | 0 | 3.182610 | 0.786267 | 0.094010 |
| 24 | 1 | 0 | 0.966844 | 1.611382 | 1.195836 |
| 25 | 1 | 0 | -2.083504 | -3.266829 | 0.784674 |
| 26 | 1 | 0 | -2.996137 | -3.650107 | -1.523343 |
| 27 | 1 | 0 | -2.662588 | -1.892849 | -3.262883 |
| 28 | 1 | 0 | -1.480923 | 0.205663 | -2.702665 |
| 29 | 1 | 0 | 1.544685 | 2.085255 | -1.971040 |
| 30 | 1 | 0 | 0.438628 | 3.466013 | -2.519356 |
| 31 | 1 | 0 | -1.927890 | 3.707432 | -0.937569 |
| 32 | 1 | 0 | -2.597363 | 2.154738 | -1.519748 |
| 33 | 1 | 0 | 0.575160 | -0.874531 | 3.756683 |
| 34 | 1 | 0 | 2.050874 | -0.306948 | 2.919618 |
| 35 | 1 | 0 | 0.856957 | 0.874376 | 3.515836 |
| 36 | 1 | 0 | 4.314374 | -1.816786 | -1.727972 |
| 37 | 1 | 0 | 5.233737 | 0.431203 | -0.691738 |
| 38 | 1 | 0 | 3.336000 | 1.736746 | 0.596388 |
| 39 | 1 | 0 | -0.308706 | 3.406267 | 1.241062 |
| 40 | 1 | 0 | -0.965823 | 2.306565 | 2.495807 |
| 41 | 1 | 0 | -1.974736 | 3.593811 | 1.827152 |

------------------------------------------------------------------------

Stoichiometry C19H18NO2S(1-)
Framework group C1[X(C19H18NO2S)]
Deg. of freedom 117
Full point group C1 NOp 1
Largest Abelian subgroup C1 NOp 1
Largest concise Abelian subgroup C1 NOp 1

After the bond distance constraint was released, the 4-membered ring broke open; the optimization did not fully complete, but below is the structure at the end of 81 optimization steps.

Input orientation:

| Center Number | Atomic Number | Atomic Type | Coordinates (Angstroms) X | Y | Z |
|---|---|---|---|---|---|
| 1 | 6 | 0 | 0.290427 | -1.655541 | 1.515250 |
| 2 | 8 | 0 | 0.476240 | -2.574292 | 2.331012 |
| 3 | 7 | 0 | 0.813198 | -0.393940 | 1.737360 |
| 4 | 6 | 0 | 0.816147 | 0.668715 | 0.714023 |
| 5 | 6 | 0 | -0.422847 | 0.631579 | -0.155339 |
| 6 | 6 | 0 | -0.900494 | -0.667252 | -0.497198 |
| 7 | 6 | 0 | -0.515541 | -1.810236 | 0.293138 |
| 8 | 6 | 0 | -0.952686 | -3.103651 | -0.023161 |
| 9 | 6 | 0 | -1.766041 | -3.351415 | -1.119344 |
| 10 | 6 | 0 | -2.138565 | -2.256720 | -1.922941 |
| 11 | 6 | 0 | -1.720872 | -0.972071 | -1.632777 |
| 12 | 6 | 0 | -0.947297 | 1.912397 | -0.570559 |
| 13 | 6 | 0 | -0.248088 | 3.088829 | -0.519671 |
| 14 | 6 | 0 | -3.391377 | 1.582991 | 0.185981 |
| 15 | 8 | 0 | -4.428511 | 0.966235 | -0.082321 |
| 16 | 6 | 0 | -2.431786 | 2.045963 | -0.912046 |
| 17 | 6 | 0 | -3.093664 | 2.013480 | 1.618550 |
| 18 | 6 | 0 | 1.640442 | -0.179743 | 2.907028 |
| 19 | 6 | 0 | 2.153597 | 0.681767 | -0.038798 |
| 20 | 16 | 0 | 2.485792 | -0.391162 | -1.381515 |
| 21 | 6 | 0 | 4.092532 | 0.241569 | -1.570598 |
| 22 | 6 | 0 | 4.354357 | 1.229131 | -0.661939 |
| 23 | 6 | 0 | 3.247241 | 1.475098 | 0.207809 |
| 24 | 1 | 0 | 0.811805 | 1.619406 | 1.268493 |
| 25 | 1 | 0 | -0.632830 | -3.904590 | 0.637552 |
| 26 | 1 | 0 | -2.096988 | -4.359367 | -1.357683 |
| 27 | 1 | 0 | -2.750376 | -2.420811 | -2.809896 |
| 28 | 1 | 0 | -1.976718 | -0.179026 | -2.324901 |
| 29 | 1 | 0 | 0.820465 | 3.129811 | -0.336806 |
| 30 | 1 | 0 | -0.740330 | 4.041450 | -0.699230 |
| 31 | 1 | 0 | -2.650418 | 3.110735 | -1.086323 |
| 32 | 1 | 0 | -2.732157 | 1.523284 | -1.822144 |
| 33 | 1 | 0 | 1.509547 | -1.035175 | 3.569733 |
| 34 | 1 | 0 | 2.704698 | -0.090027 | 2.643384 |
| 35 | 1 | 0 | 1.338905 | 0.744693 | 3.420278 |
| 36 | 1 | 0 | 4.742749 | -0.143673 | -2.345834 |
| 37 | 1 | 0 | 5.296328 | 1.767953 | -0.612112 |
| 38 | 1 | 0 | 3.250301 | 2.226645 | 0.992328 |
| 39 | 1 | 0 | -2.027191 | 2.163688 | 1.792199 |
| 40 | 1 | 0 | -3.496713 | 1.270631 | 2.311932 |
| 41 | 1 | 0 | -3.610887 | 2.965663 | 1.807772 |

Stoichiometry C19H18NO2S(1-)
Framework group C1[X(C19H18NO2S)]
Deg. of freedom 117
Full point group C1 NOp 1
Largest Abelian subgroup C1 NOp 1
Largest concise Abelian subgroup C1 NOp 1

Zero-point correction= 0.323256 (Hartree/Particle)
Thermal correction to Energy= 0.343864
Thermal correction to Enthalpy= 0.344808
Thermal correction to Gibbs Free Energy= 0.272272
Sum of electronic and zero-point Energies= -1337.928590
Sum of electronic and thermal Energies= -1337.907983
Sum of electronic and thermal Enthalpies= -1337.907039
Sum of electronic and thermal Free Energies= -1337.979574

B3LYP/6-31G(d) fopt freq

---

Simplified model of lactone structure **19b**, N-isobutyl changed to N-methyl, O changed to CH2, NEt3 changed to tBu, alkene truncated (changes and scissile bond indicated in red)

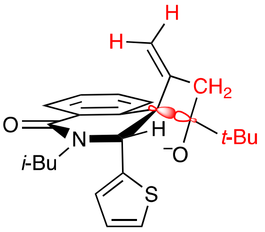


---

Input orientation:

| Center Number | Atomic Number | Atomic Type | Coordinates (Angstroms) X | Y | Z |
|---|---|---|---|---|---|
| 1 | 6 | 0 | -0.213810 | -2.122650 | 0.893118 |
| 2 | 8 | 0 | -0.212749 | -3.295262 | 1.286823 |
| 3 | 7 | 0 | 0.526532 | -1.144992 | 1.510056 |
| 4 | 6 | 0 | 0.623479 | 0.221013 | 0.935657 |
| 5 | 6 | 0 | -0.744643 | 0.717970 | 0.453795 |
| 6 | 6 | 0 | -1.305533 | -0.308438 | -0.512751 |
| 7 | 6 | 0 | -1.037411 | -1.674944 | -0.271444 |
| 8 | 6 | 0 | -1.575992 | -2.668034 | -1.097877 |
| 9 | 6 | 0 | -2.372307 | -2.321472 | -2.184970 |
| 10 | 6 | 0 | -2.623156 | -0.970578 | -2.443962 |
| 11 | 6 | 0 | -2.103130 | 0.027613 | -1.619299 |
| 12 | 6 | 0 | -1.768533 | 1.079952 | 1.526787 |
| 13 | 6 | 0 | -1.975542 | 0.617570 | 2.761951 |
| 14 | 6 | 0 | -0.957461 | 2.392905 | -0.049459 |
| 15 | 8 | 0 | -1.006878 | 2.676072 | -1.318869 |
| 16 | 6 | 0 | -2.294866 | 2.295726 | 0.812356 |
| 17 | 6 | 0 | 0.063806 | 3.256459 | 0.744701 |
| 18 | 6 | 0 | 1.296568 | -1.481181 | 2.694132 |
| 19 | 6 | 0 | 1.665335 | 0.299085 | -0.175372 |
| 20 | 16 | 0 | 3.220395 | -0.519968 | -0.027683 |
| 21 | 6 | 0 | 3.752340 | 0.094743 | -1.563762 |
| 22 | 6 | 0 | 2.791134 | 0.875016 | -2.140801 |
| 23 | 6 | 0 | 1.601255 | 0.994944 | -1.354666 |
| 24 | 1 | 0 | 0.945244 | 0.859119 | 1.766774 |
| 25 | 1 | 0 | -1.357549 | -3.704178 | -0.857930 |
| 26 | 1 | 0 | -2.788073 | -3.094693 | -2.828307 |
| 27 | 1 | 0 | -3.225855 | -0.688301 | -3.306058 |
| 28 | 1 | 0 | -2.223319 | 1.085761 | -1.841110 |
| 29 | 1 | 0 | -2.695257 | 1.095120 | 3.426401 |
| 30 | 1 | 0 | -1.450997 | -0.248874 | 3.156396 |
| 31 | 1 | 0 | -3.136476 | 2.060238 | 0.149300 |
| 32 | 1 | 0 | -2.569123 | 3.160920 | 1.433198 |
| 33 | 1 | 0 | 1.157489 | -0.706595 | 3.458673 |
| 34 | 1 | 0 | 0.938260 | -2.441713 | 3.066819 |
| 35 | 1 | 0 | 2.371458 | -1.570384 | 2.482912 |
| 36 | 1 | 0 | 4.728716 | -0.171975 | -1.947825 |
| 37 | 1 | 0 | 2.914046 | 1.352422 | -3.108607 |
| 38 | 1 | 0 | 0.729523 | 1.599811 | -1.632130 |
| 39 | 1 | 0 | 0.094370 | 3.044261 | 1.823389 |
| 40 | 1 | 0 | 1.071228 | 3.142562 | 0.330981 |
| 41 | 1 | 0 | -0.231401 | 4.305692 | 0.611987 |

Stoichiometry    C19H18NO2S(1-)
Framework group  C1[X(C19H18NO2S)]
Deg. of freedom   117
Full point group   C1   NOp  1
Largest Abelian subgroup   C1   NOp  1
Largest concise Abelian subgroup C1   NOp  1

Zero-point correction=   0.324126 (Hartree/Particle)

| | |
|---|---|
| Thermal correction to Energy= | 0.344459 |
| Thermal correction to Enthalpy= | 0.345403 |
| Thermal correction to Gibbs Free Energy= | 0.276016 |
| Sum of electronic and zero-point Energies= | -1337.895500 |
| Sum of electronic and thermal Energies= | -1337.875167 |
| Sum of electronic and thermal Enthalpies= | -1337.874223 |
| Sum of electronic and thermal Free Energies= | -1337.943610 |

Tables SI-1. Crystal data and structure refinement for major lactone **12**.

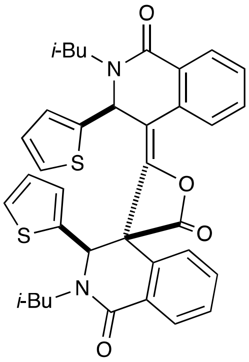


**12**

| | | |
|---|---|---|
| Identification code | 11c15a_P21n | |
| Empirical formula | C38.25 H38.75 N2 O4.75 S2 | |
| Formula weight | 666.58 | |
| Temperature | 120(2) K | |
| Wavelength | 0.71073 Å | |
| Crystal system | Monoclinic | |
| Space group | $P2_1/n$ | |
| Unit cell dimensions | a = 10.2939(14) Å | $\alpha$= 90°. |
| | b = 24.841(4) Å | $\beta$= 98.938(3)°. |
| | c = 13.7811(19) Å | $\gamma$ = 90°. |
| Volume | 3481.2(8) $Å^3$ | |
| Z | 4 | |
| Density (calculated) | 1.272 $Mg/m^3$ | |
| Absorption coefficient | 0.198 $mm^{-1}$ | |
| F(000) | 1409 | |
| Crystal size | 0.450 x 0.310 x 0.210 $mm^3$ | |
| Theta range for data collection | 2.164 to 28.281°. | |
| Index ranges | -13<=h<=13, -33<=k<=33, -18<=l<=18 | |
| Reflections collected | 37579 | |
| Independent reflections | 8645 [R(int) = 0.0520] | |
| Completeness to theta = 25.242° | 100.0 % | |
| Absorption correction | Semi-empirical from equivalents | |
| Max. and min. transmission | 0.4343 and 0.3804 | |
| Refinement method | Full-matrix least-squares on $F^2$ | |
| Data / restraints / parameters | 8645 / 951 / 493 | |

| | |
|---|---|
| Goodness-of-fit on $F^2$ | 1.105 |
| Final R indices [I>2sigma(I)] | R1 = 0.0515, wR2 = 0.1283 |
| R indices (all data) | R1 = 0.0582, wR2 = 0.1326 |
| Extinction coefficient | n/a |
| Largest diff. peak and hole | 0.561 and -0.319 e.Å$^{-3}$ |

Table. Atomic coordinates ( x $10^4$) and equivalent isotropic displacement parameters ($Å^2$x $10^3$) for compound 12. U(eq) is defined as one third of the trace of the orthogonalized $U^{ij}$ tensor.

| | x | y | z | U(eq) |
|---|---|---|---|---|
| S(1) | 3710(1) | 4819(1) | 6056(1) | 26(1) |
| S(2) | 2252(1) | 1634(1) | 3988(1) | 31(1) |
| O(1) | 4270(1) | 3839(1) | 3470(1) | 26(1) |
| O(2) | 1037(1) | 4066(1) | 7083(1) | 24(1) |
| O(3) | -1784(1) | 2149(1) | 2709(1) | 32(1) |
| O(4) | 744(1) | 3217(1) | 6420(1) | 19(1) |
| N(1) | 4040(1) | 3593(1) | 5021(1) | 18(1) |
| N(2) | 82(1) | 2630(1) | 3195(1) | 21(1) |
| C(1) | 3395(1) | 3676(1) | 5884(1) | 16(1) |
| C(2) | 1885(1) | 3689(1) | 5568(1) | 16(1) |
| C(3) | 1504(1) | 4077(1) | 4733(1) | 17(1) |
| C(4) | 283(2) | 4332(1) | 4570(1) | 22(1) |
| C(5) | -5(2) | 4698(1) | 3806(1) | 25(1) |
| C(6) | 913(2) | 4812(1) | 3201(1) | 26(1) |
| C(7) | 2114(2) | 4548(1) | 3333(1) | 23(1) |
| C(8) | 2413(1) | 4178(1) | 4098(1) | 19(1) |
| C(9) | 3668(1) | 3865(1) | 4170(1) | 19(1) |
| C(10) | 3880(1) | 4166(1) | 6483(1) | 19(1) |
| C(11) | 4486(2) | 4157(1) | 7439(1) | 28(1) |
| C(12) | 4817(2) | 4680(1) | 7818(2) | 40(1) |
| C(13) | 4456(2) | 5072(1) | 7159(2) | 35(1) |
| C(14) | 1204(1) | 3742(1) | 6486(1) | 18(1) |
| C(15) | 5181(1) | 3228(1) | 5115(1) | 21(1) |
| C(16) | 6402(2) | 3407(1) | 5811(1) | 25(1) |
| C(17) | 6864(2) | 3966(1) | 5562(2) | 36(1) |
| C(18) | 7476(2) | 2989(1) | 5739(2) | 33(1) |
| C(19) | 1294(1) | 2714(1) | 3907(1) | 18(1) |
| C(20) | 923(1) | 2722(1) | 4925(1) | 17(1) |
| C(21) | 114(2) | 2263(1) | 5147(1) | 20(1) |
| C(22) | 175(2) | 2045(1) | 6086(1) | 25(1) |
| C(23) | -596(2) | 1602(1) | 6230(1) | 32(1) |

| | | | | |
|---|---|---|---|---|
| C(24) | -1433(2) | 1380(1) | 5450(2) | 37(1) |
| C(25) | -1522(2) | 1598(1) | 4519(2) | 31(1) |
| C(26) | -736(2) | 2037(1) | 4361(1) | 23(1) |
| C(27) | -863(2) | 2273(1) | 3355(1) | 23(1) |
| C(28) | 2354(2) | 2315(1) | 3759(1) | 22(1) |
| C(29) | 3483(2) | 2433(1) | 3400(1) | 30(1) |
| C(30) | 4284(2) | 1973(1) | 3320(1) | 37(1) |
| C(31) | 3738(2) | 1516(1) | 3597(1) | 37(1) |
| C(32) | 1214(1) | 3140(1) | 5515(1) | 17(1) |
| C(33) | 76(2) | 2871(1) | 2228(1) | 26(1) |
| C(34) | -236(2) | 3472(1) | 2210(1) | 30(1) |
| C(35) | -1645(2) | 3577(1) | 2329(2) | 49(1) |
| C(36) | 95(3) | 3712(1) | 1263(2) | 58(1) |
| C(42) | 2017(11) | 4846(6) | -807(11) | 67(2) |
| C(43) | 1251(10) | 5021(6) | -24(9) | 52(1) |
| O(44) | -62(6) | 4934(2) | -292(4) | 44(1) |
| C(45) | -839(8) | 5115(4) | 339(7) | 45(1) |
| C(46) | -2218(8) | 4895(3) | -46(6) | 57(2) |
| O(47) | 1616(4) | 5205(2) | 755(3) | 51(1) |
| C(51) | 3066(18) | 5111(11) | -641(18) | 68(3) |
| C(52) | 1760(20) | 4789(16) | -780(30) | 67(2) |
| C(53) | 977(19) | 4974(19) | 0(20) | 52(1) |
| C(54) | -473(18) | 4886(14) | -270(20) | 49(2) |
| C(55) | -1221(18) | 5112(11) | 490(18) | 45(1) |
| C(56) | -2643(16) | 4902(10) | 309(17) | 50(3) |

Table. Bond lengths [Å] and angles [°] for compound 12.

| | | | |
|---|---|---|---|
| S(1)-C(13) | 1.712(2) | C(11)-H(11) | 0.9500 |
| S(1)-C(10) | 1.7265(16) | C(12)-C(13) | 1.345(3) |
| S(2)-C(31) | 1.725(2) | C(12)-H(12) | 0.9500 |
| S(2)-C(28) | 1.7274(17) | C(13)-H(13) | 0.9500 |
| O(1)-C(9) | 1.2279(18) | C(14)-C(32) | 2.008(2) |
| O(2)-C(14) | 1.1817(18) | C(15)-C(16) | 1.525(2) |
| O(3)-C(27) | 1.234(2) | C(15)-H(15A) | 0.9900 |
| O(4)-C(14) | 1.3853(18) | C(15)-H(15B) | 0.9900 |
| O(4)-C(32) | 1.4187(17) | C(16)-C(17) | 1.525(2) |
| N(1)-C(9) | 1.3558(19) | C(16)-C(18) | 1.531(2) |
| N(1)-C(1) | 1.4646(18) | C(16)-H(16) | 1.0000 |
| N(1)-C(15) | 1.4716(18) | C(17)-H(17A) | 0.9800 |
| N(2)-C(27) | 1.358(2) | C(17)-H(17B) | 0.9800 |
| N(2)-C(33) | 1.460(2) | C(17)-H(17C) | 0.9800 |
| N(2)-C(19) | 1.4781(19) | C(18)-H(18A) | 0.9800 |
| C(1)-C(10) | 1.510(2) | C(18)-H(18B) | 0.9800 |
| C(1)-C(2) | 1.5484(19) | C(18)-H(18C) | 0.9800 |
| C(1)-H(1) | 1.0000 | C(19)-C(28) | 1.510(2) |
| C(2)-C(3) | 1.506(2) | C(19)-C(20) | 1.511(2) |
| C(2)-C(32) | 1.526(2) | C(19)-H(19) | 1.0000 |
| C(2)-C(14) | 1.545(2) | C(20)-C(32) | 1.323(2) |
| C(3)-C(4) | 1.393(2) | C(20)-C(21) | 1.473(2) |
| C(3)-C(8) | 1.400(2) | C(21)-C(22) | 1.395(2) |
| C(4)-C(5) | 1.389(2) | C(21)-C(26) | 1.400(2) |
| C(4)-H(4) | 0.9500 | C(22)-C(23) | 1.388(2) |
| C(5)-C(6) | 1.382(3) | C(22)-H(22) | 0.9500 |
| C(5)-H(5) | 0.9500 | C(23)-C(24) | 1.383(3) |
| C(6)-C(7) | 1.386(2) | C(23)-H(23) | 0.9500 |
| C(6)-H(6) | 0.9500 | C(24)-C(25) | 1.383(3) |
| C(7)-C(8) | 1.396(2) | C(24)-H(24) | 0.9500 |
| C(7)-H(7) | 0.9500 | C(25)-C(26) | 1.396(2) |
| C(8)-C(9) | 1.498(2) | C(25)-H(25) | 0.9500 |
| C(10)-C(11) | 1.368(2) | C(26)-C(27) | 1.492(2) |
| C(11)-C(12) | 1.421(3) | C(28)-C(29) | 1.364(2) |

| | |
|---|---|
| C(29)-C(30) | 1.424(3) |
| C(29)-H(29) | 0.9500 |
| C(30)-C(31) | 1.347(3) |
| C(30)-H(30) | 0.9500 |
| C(31)-H(31) | 0.9500 |
| C(33)-C(34) | 1.527(2) |
| C(33)-H(33A) | 0.9900 |
| C(33)-H(33B) | 0.9900 |
| C(34)-C(35) | 1.508(3) |
| C(34)-C(36) | 1.520(3) |
| C(34)-H(34) | 1.0000 |
| C(35)-H(35A) | 0.9800 |
| C(35)-H(35B) | 0.9800 |
| C(35)-H(35C) | 0.9800 |
| C(36)-H(36A) | 0.9800 |
| C(36)-H(36B) | 0.9800 |
| C(36)-H(36C) | 0.9800 |
| C(42)-C(43) | 1.496(10) |
| C(42)-H(42A) | 0.9596 |
| C(42)-H(42B) | 0.9618 |
| C(42)-H(42C) | 0.9582 |
| C(43)-O(47) | 1.175(13) |
| C(43)-O(44) | 1.362(11) |
| O(44)-C(45) | 1.347(11) |
| C(45)-C(46) | 1.537(8) |
| C(45)-H(45A) | 0.9700 |
| C(45)-H(45B) | 0.9705 |
| C(46)-H(46A) | 0.9589 |
| C(46)-H(46B) | 0.9602 |
| C(46)-H(46C) | 0.9604 |
| C(51)-C(52) | 1.555(14) |
| C(51)-H(51A) | 0.9595 |
| C(51)-H(51B) | 0.9632 |
| C(51)-H(51C) | 0.9612 |
| C(52)-C(53) | 1.506(14) |
| C(52)-H(52A) | 0.9635 |
| C(52)-H(52B) | 0.9731 |
| C(53)-C(54) | 1.498(13) |
| C(53)-H(53A) | 0.9717 |
| C(53)-H(53B) | 0.9694 |
| C(54)-C(55) | 1.505(13) |
| C(54)-H(54A) | 0.9692 |
| C(54)-H(54B) | 0.9696 |
| C(55)-C(56) | 1.537(13) |
| C(55)-H(55A) | 0.9724 |
| C(55)-H(55B) | 0.9695 |
| C(56)-H(56A) | 0.9589 |
| C(56)-H(56B) | 0.9601 |
| C(56)-H(56C) | 0.9572 |

| | |
|---|---|
| C(13)-S(1)-C(10) | 91.99(9) |
| C(31)-S(2)-C(28) | 91.58(9) |
| C(14)-O(4)-C(32) | 91.43(10) |
| C(9)-N(1)-C(1) | 121.88(12) |
| C(9)-N(1)-C(15) | 119.83(12) |
| C(1)-N(1)-C(15) | 118.16(12) |
| C(27)-N(2)-C(33) | 120.99(13) |
| C(27)-N(2)-C(19) | 122.48(13) |
| C(33)-N(2)-C(19) | 115.47(13) |
| N(1)-C(1)-C(10) | 113.88(12) |
| N(1)-C(1)-C(2) | 109.65(11) |
| C(10)-C(1)-C(2) | 111.71(12) |
| N(1)-C(1)-H(1) | 107.1 |
| C(10)-C(1)-H(1) | 107.1 |
| C(2)-C(1)-H(1) | 107.1 |
| C(3)-C(2)-C(32) | 118.30(12) |
| C(3)-C(2)-C(14) | 118.35(12) |
| C(32)-C(2)-C(14) | 81.64(10) |
| C(3)-C(2)-C(1) | 111.07(12) |
| C(32)-C(2)-C(1) | 114.97(12) |

| | | | |
|---|---|---|---|
| C(14)-C(2)-C(1) | 109.52(11) | O(4)-C(14)-C(2) | 93.47(11) |
| C(4)-C(3)-C(8) | 119.55(14) | O(2)-C(14)-C(32) | 170.75(13) |
| C(4)-C(3)-C(2) | 122.29(13) | O(4)-C(14)-C(32) | 44.95(7) |
| C(8)-C(3)-C(2) | 118.15(13) | C(2)-C(14)-C(32) | 48.78(8) |
| C(5)-C(4)-C(3) | 119.95(15) | N(1)-C(15)-C(16) | 116.41(13) |
| C(5)-C(4)-H(4) | 120.0 | N(1)-C(15)-H(15A) | 108.2 |
| C(3)-C(4)-H(4) | 120.0 | C(16)-C(15)-H(15A) | 108.2 |
| C(6)-C(5)-C(4) | 120.42(15) | N(1)-C(15)-H(15B) | 108.2 |
| C(6)-C(5)-H(5) | 119.8 | C(16)-C(15)-H(15B) | 108.2 |
| C(4)-C(5)-H(5) | 119.8 | H(15A)-C(15)-H(15B) | 107.3 |
| C(5)-C(6)-C(7) | 120.23(15) | C(17)-C(16)-C(15) | 112.28(14) |
| C(5)-C(6)-H(6) | 119.9 | C(17)-C(16)-C(18) | 110.49(14) |
| C(7)-C(6)-H(6) | 119.9 | C(15)-C(16)-C(18) | 107.11(14) |
| C(6)-C(7)-C(8) | 119.85(15) | C(17)-C(16)-H(16) | 109.0 |
| C(6)-C(7)-H(7) | 120.1 | C(15)-C(16)-H(16) | 109.0 |
| C(8)-C(7)-H(7) | 120.1 | C(18)-C(16)-H(16) | 109.0 |
| C(7)-C(8)-C(3) | 119.93(14) | C(16)-C(17)-H(17A) | 109.5 |
| C(7)-C(8)-C(9) | 118.64(13) | C(16)-C(17)-H(17B) | 109.5 |
| C(3)-C(8)-C(9) | 121.25(13) | H(17A)-C(17)-H(17B) | 109.5 |
| O(1)-C(9)-N(1) | 122.96(14) | C(16)-C(17)-H(17C) | 109.5 |
| O(1)-C(9)-C(8) | 120.70(13) | H(17A)-C(17)-H(17C) | 109.5 |
| N(1)-C(9)-C(8) | 116.24(13) | H(17B)-C(17)-H(17C) | 109.5 |
| C(11)-C(10)-C(1) | 125.18(14) | C(16)-C(18)-H(18A) | 109.5 |
| C(11)-C(10)-S(1) | 110.51(12) | C(16)-C(18)-H(18B) | 109.5 |
| C(1)-C(10)-S(1) | 124.29(11) | H(18A)-C(18)-H(18B) | 109.5 |
| C(10)-C(11)-C(12) | 112.73(16) | C(16)-C(18)-H(18C) | 109.5 |
| C(10)-C(11)-H(11) | 123.6 | H(18A)-C(18)-H(18C) | 109.5 |
| C(12)-C(11)-H(11) | 123.6 | H(18B)-C(18)-H(18C) | 109.5 |
| C(13)-C(12)-C(11) | 112.89(17) | N(2)-C(19)-C(28) | 111.73(12) |
| C(13)-C(12)-H(12) | 123.6 | N(2)-C(19)-C(20) | 107.98(12) |
| C(11)-C(12)-H(12) | 123.6 | C(28)-C(19)-C(20) | 115.20(13) |
| C(12)-C(13)-S(1) | 111.87(14) | N(2)-C(19)-H(19) | 107.2 |
| C(12)-C(13)-H(13) | 124.1 | C(28)-C(19)-H(19) | 107.2 |
| S(1)-C(13)-H(13) | 124.1 | C(20)-C(19)-H(19) | 107.2 |
| O(2)-C(14)-O(4) | 126.96(14) | C(32)-C(20)-C(21) | 124.13(13) |
| O(2)-C(14)-C(2) | 139.56(14) | C(32)-C(20)-C(19) | 121.01(13) |

| | | | |
|---|---|---|---|
| C(21)-C(20)-C(19) | 114.57(13) | O(4)-C(32)-C(2) | 92.96(11) |
| C(22)-C(21)-C(26) | 119.68(15) | C(20)-C(32)-C(14) | 165.77(12) |
| C(22)-C(21)-C(20) | 123.25(14) | O(4)-C(32)-C(14) | 43.62(7) |
| C(26)-C(21)-C(20) | 117.07(14) | C(2)-C(32)-C(14) | 49.59(8) |
| C(23)-C(22)-C(21) | 119.71(16) | N(2)-C(33)-C(34) | 112.82(14) |
| C(23)-C(22)-H(22) | 120.1 | N(2)-C(33)-H(33A) | 109.0 |
| C(21)-C(22)-H(22) | 120.1 | C(34)-C(33)-H(33A) | 109.0 |
| C(24)-C(23)-C(22) | 120.39(17) | N(2)-C(33)-H(33B) | 109.0 |
| C(24)-C(23)-H(23) | 119.8 | C(34)-C(33)-H(33B) | 109.0 |
| C(22)-C(23)-H(23) | 119.8 | H(33A)-C(33)-H(33B) | 107.8 |
| C(23)-C(24)-C(25) | 120.61(17) | C(35)-C(34)-C(36) | 111.89(19) |
| C(23)-C(24)-H(24) | 119.7 | C(35)-C(34)-C(33) | 111.77(16) |
| C(25)-C(24)-H(24) | 119.7 | C(36)-C(34)-C(33) | 108.88(18) |
| C(24)-C(25)-C(26) | 119.57(17) | C(35)-C(34)-H(34) | 108.1 |
| C(24)-C(25)-H(25) | 120.2 | C(36)-C(34)-H(34) | 108.1 |
| C(26)-C(25)-H(25) | 120.2 | C(33)-C(34)-H(34) | 108.1 |
| C(25)-C(26)-C(21) | 120.02(16) | C(34)-C(35)-H(35A) | 109.5 |
| C(25)-C(26)-C(27) | 118.87(15) | C(34)-C(35)-H(35B) | 109.5 |
| C(21)-C(26)-C(27) | 121.05(14) | H(35A)-C(35)-H(35B) | 109.5 |
| O(3)-C(27)-N(2) | 122.30(15) | C(34)-C(35)-H(35C) | 109.5 |
| O(3)-C(27)-C(26) | 121.22(15) | H(35A)-C(35)-H(35C) | 109.5 |
| N(2)-C(27)-C(26) | 116.49(14) | H(35B)-C(35)-H(35C) | 109.5 |
| C(29)-C(28)-C(19) | 125.59(15) | C(34)-C(36)-H(36A) | 109.5 |
| C(29)-C(28)-S(2) | 110.82(13) | C(34)-C(36)-H(36B) | 109.5 |
| C(19)-C(28)-S(2) | 123.55(12) | H(36A)-C(36)-H(36B) | 109.5 |
| C(28)-C(29)-C(30) | 113.10(18) | C(34)-C(36)-H(36C) | 109.5 |
| C(28)-C(29)-H(29) | 123.4 | H(36A)-C(36)-H(36C) | 109.5 |
| C(30)-C(29)-H(29) | 123.4 | H(36B)-C(36)-H(36C) | 109.5 |
| C(31)-C(30)-C(29) | 112.48(17) | C(43)-C(42)-H(42A) | 109.4 |
| C(31)-C(30)-H(30) | 123.8 | C(43)-C(42)-H(42B) | 109.4 |
| C(29)-C(30)-H(30) | 123.8 | H(42A)-C(42)-H(42B) | 109.4 |
| C(30)-C(31)-S(2) | 111.99(14) | C(43)-C(42)-H(42C) | 109.6 |
| C(30)-C(31)-H(31) | 124.0 | H(42A)-C(42)-H(42C) | 109.7 |
| S(2)-C(31)-H(31) | 124.0 | H(42B)-C(42)-H(42C) | 109.5 |
| C(20)-C(32)-O(4) | 124.77(13) | O(47)-C(43)-O(44) | 118.2(8) |
| C(20)-C(32)-C(2) | 141.96(13) | O(47)-C(43)-C(42) | 130.0(9) |

| | | | |
|---|---|---|---|
| O(44)-C(43)-C(42) | 111.8(8) | H(52A)-C(52)-H(52B) | 108.8 |
| C(45)-O(44)-C(43) | 115.9(6) | C(54)-C(53)-C(52) | 113.3(14) |
| O(44)-C(45)-C(46) | 105.5(7) | C(54)-C(53)-H(53A) | 109.0 |
| O(44)-C(45)-H(45A) | 110.6 | C(52)-C(53)-H(53A) | 109.0 |
| C(46)-C(45)-H(45A) | 110.7 | C(54)-C(53)-H(53B) | 109.1 |
| O(44)-C(45)-H(45B) | 110.6 | C(52)-C(53)-H(53B) | 108.6 |
| C(46)-C(45)-H(45B) | 110.6 | H(53A)-C(53)-H(53B) | 107.8 |
| H(45A)-C(45)-H(45B) | 108.8 | C(53)-C(54)-C(55) | 111.6(13) |
| C(45)-C(46)-H(46A) | 109.5 | C(53)-C(54)-H(54A) | 109.1 |
| C(45)-C(46)-H(46B) | 109.4 | C(55)-C(54)-H(54A) | 109.3 |
| H(46A)-C(46)-H(46B) | 109.5 | C(53)-C(54)-H(54B) | 109.2 |
| C(45)-C(46)-H(46C) | 109.4 | C(55)-C(54)-H(54B) | 109.5 |
| H(46A)-C(46)-H(46C) | 109.5 | H(54A)-C(54)-H(54B) | 108.1 |
| H(46B)-C(46)-H(46C) | 109.4 | C(54)-C(55)-C(56) | 109.7(12) |
| C(52)-C(51)-H(51A) | 109.8 | C(54)-C(55)-H(55A) | 109.5 |
| C(52)-C(51)-H(51B) | 109.8 | C(56)-C(55)-H(55A) | 109.8 |
| H(51A)-C(51)-H(51B) | 109.2 | C(54)-C(55)-H(55B) | 109.7 |
| C(52)-C(51)-H(51C) | 109.5 | C(56)-C(55)-H(55B) | 109.9 |
| H(51A)-C(51)-H(51C) | 109.4 | H(55A)-C(55)-H(55B) | 108.1 |
| H(51B)-C(51)-H(51C) | 109.1 | C(55)-C(56)-H(56A) | 109.2 |
| C(53)-C(52)-C(51) | 107.7(13) | C(55)-C(56)-H(56B) | 109.2 |
| C(53)-C(52)-H(52A) | 110.6 | H(56A)-C(56)-H(56B) | 109.5 |
| C(51)-C(52)-H(52A) | 110.1 | C(55)-C(56)-H(56C) | 109.3 |
| C(53)-C(52)-H(52B) | 110.0 | H(56A)-C(56)-H(56C) | 109.8 |
| C(51)-C(52)-H(52B) | 109.6 | H(56B)-C(56)-H(56C) | 109.7 |

Table. Anisotropic displacement parameters ($Å^2$x $10^3$) for compound 12. The anisotropic displacement factor exponent takes the form: $-2\pi^2[ h^2 a^{*2}U^{11} + ... + 2 h k a^* b^* U^{12} ]$

| | $U^{11}$ | $U^{22}$ | $U^{33}$ | $U^{23}$ | $U^{13}$ | $U^{12}$ |
|---|---|---|---|---|---|---|
| S(1) | 28(1) | 19(1) | 32(1) | 1(1) | 4(1) | -2(1) |
| S(2) | 31(1) | 25(1) | 38(1) | 0(1) | 4(1) | 8(1) |
| O(1) | 27(1) | 30(1) | 22(1) | 3(1) | 11(1) | 5(1) |
| O(2) | 25(1) | 26(1) | 22(1) | -6(1) | 8(1) | 1(1) |
| O(3) | 28(1) | 33(1) | 31(1) | -7(1) | -4(1) | -6(1) |
| O(4) | 18(1) | 23(1) | 17(1) | -2(1) | 6(1) | -1(1) |
| N(1) | 15(1) | 21(1) | 19(1) | 1(1) | 4(1) | 3(1) |
| N(2) | 22(1) | 22(1) | 18(1) | -1(1) | 0(1) | -1(1) |
| C(1) | 14(1) | 19(1) | 16(1) | 1(1) | 2(1) | 0(1) |
| C(2) | 14(1) | 18(1) | 17(1) | -2(1) | 3(1) | 0(1) |
| C(3) | 17(1) | 17(1) | 16(1) | -2(1) | 1(1) | 0(1) |
| C(4) | 18(1) | 25(1) | 22(1) | -4(1) | 2(1) | 3(1) |
| C(5) | 22(1) | 24(1) | 27(1) | -3(1) | -4(1) | 6(1) |
| C(6) | 31(1) | 22(1) | 23(1) | 4(1) | -4(1) | 3(1) |
| C(7) | 25(1) | 22(1) | 21(1) | 2(1) | 2(1) | 0(1) |
| C(8) | 18(1) | 20(1) | 18(1) | -1(1) | 2(1) | 1(1) |
| C(9) | 18(1) | 20(1) | 18(1) | 1(1) | 4(1) | 0(1) |
| C(10) | 15(1) | 20(1) | 21(1) | 0(1) | 5(1) | -3(1) |
| C(11) | 28(1) | 27(1) | 27(1) | 0(1) | -1(1) | -7(1) |
| C(12) | 44(1) | 40(1) | 32(1) | -10(1) | -2(1) | -15(1) |
| C(13) | 34(1) | 28(1) | 42(1) | -10(1) | 8(1) | -13(1) |
| C(14) | 15(1) | 22(1) | 18(1) | 0(1) | 2(1) | 1(1) |
| C(15) | 16(1) | 23(1) | 25(1) | 2(1) | 4(1) | 5(1) |
| C(16) | 16(1) | 32(1) | 28(1) | 2(1) | 4(1) | 3(1) |
| C(17) | 21(1) | 33(1) | 56(1) | -1(1) | 10(1) | -4(1) |
| C(18) | 18(1) | 42(1) | 38(1) | 5(1) | 4(1) | 8(1) |
| C(19) | 19(1) | 21(1) | 17(1) | -2(1) | 3(1) | -1(1) |
| C(20) | 16(1) | 19(1) | 17(1) | 1(1) | 3(1) | 2(1) |
| C(21) | 19(1) | 18(1) | 23(1) | -2(1) | 7(1) | 2(1) |
| C(22) | 31(1) | 21(1) | 24(1) | 0(1) | 8(1) | 3(1) |
| C(23) | 47(1) | 23(1) | 32(1) | 3(1) | 18(1) | -1(1) |

| | | | | | | |
|---|---|---|---|---|---|---|
| C(24) | 46(1) | 24(1) | 45(1) | -1(1) | 21(1) | -10(1) |
| C(25) | 32(1) | 26(1) | 38(1) | -8(1) | 11(1) | -8(1) |
| C(26) | 22(1) | 19(1) | 28(1) | -4(1) | 7(1) | -2(1) |
| C(27) | 23(1) | 20(1) | 25(1) | -6(1) | 2(1) | 0(1) |
| C(28) | 23(1) | 25(1) | 16(1) | -3(1) | 2(1) | 2(1) |
| C(29) | 30(1) | 40(1) | 23(1) | -5(1) | 10(1) | 2(1) |
| C(30) | 32(1) | 54(1) | 26(1) | -5(1) | 9(1) | 13(1) |
| C(31) | 37(1) | 44(1) | 30(1) | -6(1) | 4(1) | 20(1) |
| C(32) | 13(1) | 21(1) | 16(1) | 1(1) | 3(1) | 2(1) |
| C(33) | 32(1) | 30(1) | 16(1) | -1(1) | 1(1) | -1(1) |
| C(34) | 31(1) | 29(1) | 26(1) | 5(1) | -6(1) | -5(1) |
| C(35) | 34(1) | 36(1) | 75(2) | 2(1) | -2(1) | 5(1) |
| C(36) | 74(2) | 58(2) | 39(1) | 24(1) | 1(1) | -9(1) |
| C(42) | 73(3) | 69(3) | 61(3) | 6(3) | 15(3) | 17(3) |
| C(43) | 59(3) | 50(3) | 47(2) | 9(2) | 5(2) | 14(3) |
| O(44) | 53(3) | 35(2) | 38(2) | 1(2) | -6(3) | -2(2) |
| C(45) | 47(3) | 43(2) | 43(3) | 10(2) | -2(2) | -10(3) |
| C(46) | 56(3) | 53(3) | 56(3) | 8(3) | -6(3) | -25(3) |
| O(47) | 48(2) | 55(2) | 46(2) | -12(2) | -4(2) | -2(2) |
| C(51) | 73(5) | 73(6) | 63(6) | 4(6) | 21(5) | 11(5) |
| C(52) | 73(3) | 69(3) | 61(3) | 6(3) | 15(3) | 17(3) |
| C(53) | 59(3) | 50(3) | 47(2) | 9(2) | 5(2) | 14(3) |
| C(54) | 53(4) | 45(4) | 47(4) | 9(4) | -1(4) | -4(4) |
| C(55) | 47(3) | 43(2) | 43(3) | 10(2) | -2(2) | -10(3) |
| C(56) | 48(5) | 50(5) | 50(5) | 4(5) | -4(4) | -9(5) |

Table. Hydrogen coordinates ( x $10^4$) and isotropic displacement parameters ($Å^2$x $10^3$) for compound 12.

| | x | y | z | U(eq) |
|---|---|---|---|---|
| H(1) | 3599 | 3355 | 6319 | 20 |
| H(4) | -352 | 4255 | 4983 | 26 |
| H(5) | -838 | 4871 | 3697 | 30 |
| H(6) | 719 | 5072 | 2694 | 31 |
| H(7) | 2733 | 4619 | 2905 | 27 |
| H(11) | 4667 | 3836 | 7811 | 34 |
| H(12) | 5248 | 4745 | 8468 | 48 |
| H(13) | 4598 | 5445 | 7289 | 42 |
| H(15A) | 5427 | 3177 | 4455 | 25 |
| H(15B) | 4905 | 2873 | 5338 | 25 |
| H(16) | 6200 | 3410 | 6498 | 30 |
| H(17A) | 7030 | 3971 | 4880 | 54 |
| H(17B) | 7676 | 4057 | 6003 | 54 |
| H(17C) | 6183 | 4232 | 5641 | 54 |
| H(18A) | 7732 | 3005 | 5084 | 49 |
| H(18B) | 7140 | 2629 | 5851 | 49 |
| H(18C) | 8242 | 3066 | 6236 | 49 |
| H(19) | 1633 | 3081 | 3780 | 22 |
| H(22) | 742 | 2198 | 6625 | 30 |
| H(23) | -549 | 1451 | 6867 | 39 |
| H(24) | -1951 | 1076 | 5555 | 44 |
| H(25) | -2115 | 1449 | 3989 | 37 |
| H(29) | 3712 | 2786 | 3222 | 36 |
| H(30) | 5111 | 1986 | 3097 | 44 |
| H(31) | 4126 | 1170 | 3579 | 44 |
| H(33A) | -585 | 2685 | 1745 | 32 |
| H(33B) | 948 | 2817 | 2026 | 32 |
| H(34) | 344 | 3646 | 2773 | 36 |
| H(35A) | -1794 | 3966 | 2355 | 74 |
| H(35B) | -1826 | 3411 | 2938 | 74 |

| | | | | |
|---|---|---|---|---|
| H(35C) | -2233 | 3423 | 1770 | 74 |
| H(36A) | -426 | 3533 | 700 | 87 |
| H(36B) | 1033 | 3661 | 1238 | 87 |
| H(36C) | -108 | 4098 | 1243 | 87 |
| H(42A) | 2936 | 4912 | -592 | 101 |
| H(42B) | 1881 | 4467 | -930 | 101 |
| H(42C) | 1728 | 5043 | -1398 | 101 |
| H(45A) | -846 | 5506 | 351 | 54 |
| H(45B) | -532 | 4984 | 998 | 54 |
| H(46A) | -2822 | 5012 | 376 | 85 |
| H(46B) | -2502 | 5028 | -700 | 85 |
| H(46C) | -2190 | 4509 | -57 | 85 |
| H(51A) | 3585 | 5003 | -1128 | 103 |
| H(51B) | 2881 | 5491 | -707 | 103 |
| H(51C) | 3548 | 5043 | 2 | 103 |
| H(52A) | 1935 | 4408 | -714 | 81 |
| H(52B) | 1265 | 4859 | -1427 | 81 |
| H(53A) | 1140 | 5355 | 122 | 63 |
| H(53B) | 1288 | 4782 | 604 | 63 |
| H(54A) | -644 | 4503 | -344 | 59 |
| H(54B) | -774 | 5056 | -901 | 59 |
| H(55A) | -1219 | 5503 | 456 | 54 |
| H(55B) | -796 | 5006 | 1139 | 54 |
| H(56A) | -3114 | 5049 | 796 | 76 |
| H(56B) | -3058 | 5013 | -333 | 76 |
| H(56C) | -2636 | 4518 | 349 | 76 |

Table. Torsion angles [°] for compound 12.

| | | | |
|---|---|---|---|
| C(9)-N(1)-C(1)-C(10) | -81.29(17) | C(3)-C(8)-C(9)-N(1) | -17.4(2) |
| C(15)-N(1)-C(1)-C(10) | 94.53(16) | N(1)-C(1)-C(10)-C(11) | -117.98(17) |
| C(9)-N(1)-C(1)-C(2) | 44.71(18) | C(2)-C(1)-C(10)-C(11) | 117.11(17) |
| C(15)-N(1)-C(1)-C(2) | -139.47(13) | N(1)-C(1)-C(10)-S(1) | 63.60(16) |
| N(1)-C(1)-C(2)-C(3) | -50.98(15) | C(2)-C(1)-C(10)-S(1) | -61.31(16) |
| C(10)-C(1)-C(2)-C(3) | 76.24(15) | C(13)-S(1)-C(10)-C(11) | 0.25(14) |
| N(1)-C(1)-C(2)-C(32) | 86.74(14) | C(13)-S(1)-C(10)-C(1) | 178.87(13) |
| C(10)-C(1)-C(2)-C(32) | -146.03(12) | C(1)-C(10)-C(11)-C(12) | -179.04(15) |
| N(1)-C(1)-C(2)-C(14) | 176.45(11) | S(1)-C(10)-C(11)-C(12) | -0.4(2) |
| C(10)-C(1)-C(2)-C(14) | -56.32(15) | C(10)-C(11)-C(12)-C(13) | 0.5(3) |
| C(32)-C(2)-C(3)-C(4) | 70.69(18) | C(11)-C(12)-C(13)-S(1) | -0.3(2) |
| C(14)-C(2)-C(3)-C(4) | -25.2(2) | C(10)-S(1)-C(13)-C(12) | 0.01(16) |
| C(1)-C(2)-C(3)-C(4) | -153.15(14) | C(32)-O(4)-C(14)-O(2) | 174.04(16) |
| C(32)-C(2)-C(3)-C(8) | -108.66(15) | C(32)-O(4)-C(14)-C(2) | -5.56(11) |
| C(14)-C(2)-C(3)-C(8) | 155.44(13) | C(3)-C(2)-C(14)-O(2) | -56.6(3) |
| C(1)-C(2)-C(3)-C(8) | 27.51(18) | C(32)-C(2)-C(14)-O(2) | -174.3(2) |
| C(8)-C(3)-C(4)-C(5) | -2.4(2) | C(1)-C(2)-C(14)-O(2) | 72.1(2) |
| C(2)-C(3)-C(4)-C(5) | 178.22(14) | C(3)-C(2)-C(14)-O(4) | 122.94(13) |
| C(3)-C(4)-C(5)-C(6) | 0.1(2) | C(32)-C(2)-C(14)-O(4) | 5.22(10) |
| C(4)-C(5)-C(6)-C(7) | 2.1(3) | C(1)-C(2)-C(14)-O(4) | -108.40(12) |
| C(5)-C(6)-C(7)-C(8) | -1.9(2) | C(3)-C(2)-C(14)-C(32) | 117.72(15) |
| C(6)-C(7)-C(8)-C(3) | -0.5(2) | C(1)-C(2)-C(14)-C(32) | -113.62(13) |
| C(6)-C(7)-C(8)-C(9) | 174.78(14) | C(9)-N(1)-C(15)-C(16) | 110.52(16) |
| C(4)-C(3)-C(8)-C(7) | 2.6(2) | C(1)-N(1)-C(15)-C(16) | -65.39(18) |
| C(2)-C(3)-C(8)-C(7) | -177.98(14) | N(1)-C(15)-C(16)-C(17) | -54.93(19) |
| C(4)-C(3)-C(8)-C(9) | -172.48(14) | N(1)-C(15)-C(16)-C(18) | -176.39(13) |
| C(2)-C(3)-C(8)-C(9) | 6.9(2) | C(27)-N(2)-C(19)-C(28) | 85.99(17) |
| C(1)-N(1)-C(9)-O(1) | 172.99(14) | C(33)-N(2)-C(19)-C(28) | -82.32(16) |
| C(15)-N(1)-C(9)-O(1) | -2.8(2) | C(27)-N(2)-C(19)-C(20) | -41.68(18) |
| C(1)-N(1)-C(9)-C(8) | -10.8(2) | C(33)-N(2)-C(19)-C(20) | 150.00(13) |
| C(15)-N(1)-C(9)-C(8) | 173.47(13) | N(2)-C(19)-C(20)-C(32) | -123.27(15) |
| C(7)-C(8)-C(9)-O(1) | -16.2(2) | C(28)-C(19)-C(20)-C(32) | 111.08(16) |
| C(3)-C(8)-C(9)-O(1) | 158.97(15) | N(2)-C(19)-C(20)-C(21) | 50.74(16) |
| C(7)-C(8)-C(9)-N(1) | 167.44(14) | C(28)-C(19)-C(20)-C(21) | -74.92(17) |

| | | | |
|---|---|---|---|
| C(32)-C(20)-C(21)-C(22) | -37.9(2) | C(21)-C(20)-C(32)-C(2) | -174.37(17) |
| C(19)-C(20)-C(21)-C(22) | 148.27(15) | C(19)-C(20)-C(32)-C(2) | -1.0(3) |
| C(32)-C(20)-C(21)-C(26) | 143.00(15) | C(21)-C(20)-C(32)-C(14) | -34.9(6) |
| C(19)-C(20)-C(21)-C(26) | -30.79(19) | C(19)-C(20)-C(32)-C(14) | 138.5(5) |
| C(26)-C(21)-C(22)-C(23) | 0.7(2) | C(14)-O(4)-C(32)-C(20) | -169.12(15) |
| C(20)-C(21)-C(22)-C(23) | -178.32(15) | C(14)-O(4)-C(32)-C(2) | 5.63(11) |
| C(21)-C(22)-C(23)-C(24) | -0.6(3) | C(3)-C(2)-C(32)-C(20) | 50.1(3) |
| C(22)-C(23)-C(24)-C(25) | -0.5(3) | C(14)-C(2)-C(32)-C(20) | 167.9(2) |
| C(23)-C(24)-C(25)-C(26) | 1.6(3) | C(1)-C(2)-C(32)-C(20) | -84.4(2) |
| C(24)-C(25)-C(26)-C(21) | -1.5(3) | C(3)-C(2)-C(32)-O(4) | -122.86(13) |
| C(24)-C(25)-C(26)-C(27) | -178.90(16) | C(14)-C(2)-C(32)-O(4) | -5.10(10) |
| C(22)-C(21)-C(26)-C(25) | 0.3(2) | C(1)-C(2)-C(32)-O(4) | 102.61(12) |
| C(20)-C(21)-C(26)-C(25) | 179.44(14) | C(3)-C(2)-C(32)-C(14) | -117.77(14) |
| C(22)-C(21)-C(26)-C(27) | 177.68(14) | C(1)-C(2)-C(32)-C(14) | 107.71(13) |
| C(20)-C(21)-C(26)-C(27) | -3.2(2) | C(27)-N(2)-C(33)-C(34) | 111.27(17) |
| C(33)-N(2)-C(27)-O(3) | -2.2(2) | C(19)-N(2)-C(33)-C(34) | -80.22(17) |
| C(19)-N(2)-C(27)-O(3) | -169.93(15) | N(2)-C(33)-C(34)-C(35) | -69.2(2) |
| C(33)-N(2)-C(27)-C(26) | 177.96(14) | N(2)-C(33)-C(34)-C(36) | 166.72(16) |
| C(19)-N(2)-C(27)-C(26) | 10.3(2) | O(47)-C(43)-O(44)-C(45) | 5.7(15) |
| C(25)-C(26)-C(27)-O(3) | 12.2(2) | C(42)-C(43)-O(44)-C(45) | -175.4(10) |
| C(21)-C(26)-C(27)-O(3) | -165.21(15) | C(43)-O(44)-C(45)-C(46) | -171.6(8) |
| C(25)-C(26)-C(27)-N(2) | -168.05(15) | C(51)-C(52)-C(53)-C(54) | 156(3) |
| C(21)-C(26)-C(27)-N(2) | 14.6(2) | C(52)-C(53)-C(54)-C(55) | -176(3) |
| N(2)-C(19)-C(28)-C(29) | 109.67(18) | C(53)-C(54)-C(55)-C(56) | -167(3) |
| C(20)-C(19)-C(28)-C(29) | -126.64(17) | | |
| N(2)-C(19)-C(28)-S(2) | -67.85(16) | | |
| C(20)-C(19)-C(28)-S(2) | 55.84(17) | | |
| C(31)-S(2)-C(28)-C(29) | 0.04(14) | | |
| C(31)-S(2)-C(28)-C(19) | 177.89(14) | | |
| C(19)-C(28)-C(29)-C(30) | -178.58(15) | | |
| S(2)-C(28)-C(29)-C(30) | -0.79(19) | | |
| C(28)-C(29)-C(30)-C(31) | 1.4(2) | | |
| C(29)-C(30)-C(31)-S(2) | -1.3(2) | | |
| C(28)-S(2)-C(31)-C(30) | 0.75(16) | | |
| C(21)-C(20)-C(32)-O(4) | -2.9(2) | | |
| C(19)-C(20)-C(32)-O(4) | 170.51(13) | | |

Table. Hydrogen bonds for compound 12 [Å and °].

| D-H...A | d(D-H) | d(H...A) | d(D...A) | <(DHA) |
|---|---|---|---|---|
| C(1)-H(1)...O(3)#1 | 1.00 | 2.37 | 3.2733(19) | 149.2 |
| C(13)-H(13)...O(1)#2 | 0.95 | 2.45 | 3.185(2) | 134.2 |
| C(30)-H(30)...O(4)#3 | 0.95 | 2.55 | 3.250(2) | 131.0 |

Symmetry transformations used to generate equivalent atoms:

#1 x+1/2,-y+1/2,z+1/2 #2 -x+1,-y+1,-z+1 #3 x+1/2,-y+1/2,z-1/2

Tables SI-2. Crystal data and structure refinement for purple ketone **21**.

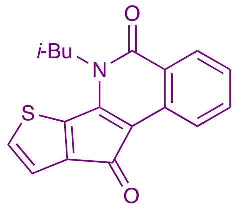


**21** (purple product)
X-ray

| | | |
|---|---|---|
| Identification code | redrod_P21n_a | |
| Empirical formula | C18 H15 N O2 S | |
| Formula weight | 309.37 | |
| Temperature | 120(2) K | |
| Wavelength | 0.71073 Å | |
| Crystal system | Monoclinic | |
| Space group | $P2_1/n$ | |
| Unit cell dimensions | a = 8.3601(8) Å | α= 90°. |
| | b = 16.3856(15) Å | β= 92.833(2)°. |
| | c = 10.5400(10) Å | γ = 90°. |
| Volume | 1442.1(2) $Å^3$ | |
| Z | 4 | |
| Density (calculated) | 1.425 $Mg/m^3$ | |
| Absorption coefficient | 0.231 $mm^{-1}$ | |
| F(000) | 648 | |
| Crystal size | 0.500 x 0.120 x 0.080 $mm^3$ | |
| Theta range for data collection | 2.300 to 31.572°. | |
| Index ranges | -12<=h<=12, -24<=k<=24, -15<=l<=15 | |
| Reflections collected | 19021 | |
| Independent reflections | 4824 [R(int) = 0.0449] | |
| Completeness to theta = 25.242° | 100.0 % | |
| Absorption correction | Semi-empirical from equivalents | |
| Max. and min. transmission | .9999 and .9240 | |
| Refinement method | Full-matrix least-squares on $F^2$ | |
| Data / restraints / parameters | 4824 / 0 / 201 | |
| Goodness-of-fit on $F^2$ | 1.236 | |
| Final R indices [I>2sigma(I)] | R1 = 0.0737, wR2 = 0.1523 | |
| R indices (all data) | R1 = 0.0846, wR2 = 0.1572 | |

| | |
|---|---|
| Extinction coefficient | n/a |
| Largest diff. peak and hole | 0.671 and -0.320 e.$Å^{-3}$ |

Table. Atomic coordinates ( x $10^4$) and equivalent isotropic displacement parameters ($Å^2$x $10^3$) for compound 21. U(eq) is defined as one third of the trace of the orthogonalized $U^{ij}$ tensor.

| | x | y | z | U(eq) |
|---|---|---|---|---|
| S(1) | 8451(1) | 3053(1) | 6971(1) | 20(1) |
| O(1) | 9411(2) | 4382(1) | 2853(1) | 22(1) |
| O(2) | 5091(2) | 6156(1) | 6972(2) | 29(1) |
| N(1) | 6620(2) | 5044(1) | 6553(2) | 18(1) |
| C(1) | 7521(2) | 4635(1) | 5719(2) | 15(1) |
| C(2) | 8259(2) | 3821(1) | 5877(2) | 16(1) |
| C(3) | 9621(3) | 2491(1) | 5989(2) | 21(1) |
| C(4) | 9854(2) | 2873(1) | 4863(2) | 19(1) |
| C(5) | 9060(2) | 3635(1) | 4806(2) | 17(1) |
| C(6) | 8859(2) | 4333(1) | 3898(2) | 16(1) |
| C(7) | 7867(2) | 4939(1) | 4550(2) | 16(1) |
| C(8) | 7282(2) | 5720(1) | 4144(2) | 17(1) |
| C(9) | 7603(2) | 6078(1) | 2966(2) | 21(1) |
| C(10) | 7017(3) | 6844(1) | 2663(2) | 25(1) |
| C(11) | 6076(3) | 7271(1) | 3504(2) | 28(1) |
| C(12) | 5721(3) | 6926(1) | 4650(2) | 25(1) |
| C(13) | 6325(2) | 6150(1) | 4988(2) | 19(1) |
| C(14) | 5948(2) | 5811(1) | 6229(2) | 20(1) |
| C(15) | 6289(2) | 4679(1) | 7791(2) | 19(1) |
| C(16) | 7650(2) | 4784(1) | 8807(2) | 18(1) |
| C(17) | 7306(3) | 4232(1) | 9931(2) | 26(1) |
| C(18) | 7852(3) | 5671(1) | 9228(2) | 22(1) |

Table. Bond lengths [Å] and angles [°] for compound 21.

| | | | |
|---|---|---|---|
| S(1)-C(2) | 1.7094(19) | C(9)-H(9) | 0.9500 |
| S(1)-C(3) | 1.725(2) | C(10)-C(11) | 1.401(3) |
| O(1)-C(6) | 1.218(2) | C(10)-H(10) | 0.9500 |
| O(2)-C(14) | 1.225(2) | C(11)-C(12) | 1.380(3) |
| N(1)-C(1) | 1.362(2) | C(11)-H(11) | 0.9500 |
| N(1)-C(14) | 1.412(3) | C(12)-C(13) | 1.407(3) |
| N(1)-C(15) | 1.474(2) | C(12)-H(12) | 0.9500 |
| C(1)-C(7) | 1.373(3) | C(13)-C(14) | 1.470(3) |
| C(1)-C(2) | 1.476(3) | C(15)-C(16) | 1.533(3) |
| C(2)-C(5) | 1.374(3) | C(15)-H(15A) | 0.9900 |
| C(3)-C(4) | 1.365(3) | C(15)-H(15B) | 0.9900 |
| C(3)-H(3) | 0.9500 | C(16)-C(18) | 1.526(3) |
| C(4)-C(5) | 1.414(3) | C(16)-C(17) | 1.529(3) |
| C(4)-H(4) | 0.9500 | C(16)-H(16) | 1.0000 |
| C(5)-C(6) | 1.496(3) | C(17)-H(17A) | 0.9800 |
| C(6)-C(7) | 1.483(3) | C(17)-H(17B) | 0.9800 |
| C(7)-C(8) | 1.428(3) | C(17)-H(17C) | 0.9800 |
| C(8)-C(9) | 1.411(3) | C(18)-H(18A) | 0.9800 |
| C(8)-C(13) | 1.414(3) | C(18)-H(18B) | 0.9800 |
| C(9)-C(10) | 1.379(3) | C(18)-H(18C) | 0.9800 |
| | | | |
| C(2)-S(1)-C(3) | 91.42(10) | C(3)-C(4)-C(5) | 110.83(18) |
| C(1)-N(1)-C(14) | 120.57(16) | C(3)-C(4)-H(4) | 124.6 |
| C(1)-N(1)-C(15) | 120.40(16) | C(5)-C(4)-H(4) | 124.6 |
| C(14)-N(1)-C(15) | 119.01(16) | C(2)-C(5)-C(4) | 114.03(17) |
| N(1)-C(1)-C(7) | 123.27(17) | C(2)-C(5)-C(6) | 108.20(17) |
| N(1)-C(1)-C(2) | 127.87(17) | C(4)-C(5)-C(6) | 137.75(18) |
| C(7)-C(1)-C(2) | 108.86(16) | O(1)-C(6)-C(7) | 128.32(18) |
| C(5)-C(2)-C(1) | 109.00(16) | O(1)-C(6)-C(5) | 126.42(18) |
| C(5)-C(2)-S(1) | 110.99(15) | C(7)-C(6)-C(5) | 105.26(15) |
| C(1)-C(2)-S(1) | 140.01(15) | C(1)-C(7)-C(8) | 120.62(17) |
| C(4)-C(3)-S(1) | 112.73(16) | C(1)-C(7)-C(6) | 108.68(16) |
| C(4)-C(3)-H(3) | 123.6 | C(8)-C(7)-C(6) | 130.70(17) |
| S(1)-C(3)-H(3) | 123.6 | C(9)-C(8)-C(13) | 118.95(18) |

| | | | |
|---|---|---|---|
| C(9)-C(8)-C(7) | 124.00(18) | C(16)-C(15)-H(15A) | 108.7 |
| C(13)-C(8)-C(7) | 117.04(17) | N(1)-C(15)-H(15B) | 108.7 |
| C(10)-C(9)-C(8) | 120.1(2) | C(16)-C(15)-H(15B) | 108.7 |
| C(10)-C(9)-H(9) | 119.9 | H(15A)-C(15)-H(15B) | 107.6 |
| C(8)-C(9)-H(9) | 119.9 | C(18)-C(16)-C(17) | 111.10(17) |
| C(9)-C(10)-C(11) | 120.8(2) | C(18)-C(16)-C(15) | 112.19(17) |
| C(9)-C(10)-H(10) | 119.6 | C(17)-C(16)-C(15) | 108.28(16) |
| C(11)-C(10)-H(10) | 119.6 | C(18)-C(16)-H(16) | 108.4 |
| C(12)-C(11)-C(10) | 120.1(2) | C(17)-C(16)-H(16) | 108.4 |
| C(12)-C(11)-H(11) | 120.0 | C(15)-C(16)-H(16) | 108.4 |
| C(10)-C(11)-H(11) | 120.0 | C(16)-C(17)-H(17A) | 109.5 |
| C(11)-C(12)-C(13) | 120.2(2) | C(16)-C(17)-H(17B) | 109.5 |
| C(11)-C(12)-H(12) | 119.9 | H(17A)-C(17)-H(17B) | 109.5 |
| C(13)-C(12)-H(12) | 119.9 | C(16)-C(17)-H(17C) | 109.5 |
| C(12)-C(13)-C(8) | 119.89(19) | H(17A)-C(17)-H(17C) | 109.5 |
| C(12)-C(13)-C(14) | 118.47(19) | H(17B)-C(17)-H(17C) | 109.5 |
| C(8)-C(13)-C(14) | 121.63(18) | C(16)-C(18)-H(18A) | 109.5 |
| O(2)-C(14)-N(1) | 119.63(19) | C(16)-C(18)-H(18B) | 109.5 |
| O(2)-C(14)-C(13) | 123.5(2) | H(18A)-C(18)-H(18B) | 109.5 |
| N(1)-C(14)-C(13) | 116.82(17) | C(16)-C(18)-H(18C) | 109.5 |
| N(1)-C(15)-C(16) | 114.07(16) | H(18A)-C(18)-H(18C) | 109.5 |
| N(1)-C(15)-H(15A) | 108.7 | H(18B)-C(18)-H(18C) | 109.5 |

Table. Anisotropic displacement parameters ($Å^2$x $10^3$) for compound 21. The anisotropic displacement factor exponent takes the form: $-2\pi^2[ h^2 a^{*2}U^{11} + ... + 2 h k a^* b^* U^{12} ]$

| | $U^{11}$ | $U^{22}$ | $U^{33}$ | $U^{23}$ | $U^{13}$ | $U^{12}$ |
|---|---|---|---|---|---|---|
| S(1) | 23(1) | 20(1) | 17(1) | 4(1) | 1(1) | -3(1) |
| O(1) | 26(1) | 26(1) | 14(1) | -1(1) | 3(1) | -1(1) |
| O(2) | 30(1) | 33(1) | 23(1) | -9(1) | 0(1) | 9(1) |
| N(1) | 18(1) | 21(1) | 14(1) | -3(1) | 1(1) | -1(1) |
| C(1) | 14(1) | 17(1) | 14(1) | -2(1) | -2(1) | -2(1) |
| C(2) | 18(1) | 16(1) | 15(1) | 2(1) | -2(1) | -4(1) |
| C(3) | 24(1) | 17(1) | 23(1) | -1(1) | -1(1) | -1(1) |
| C(4) | 22(1) | 16(1) | 19(1) | -4(1) | -1(1) | -1(1) |
| C(5) | 17(1) | 18(1) | 15(1) | -1(1) | -1(1) | -3(1) |
| C(6) | 16(1) | 18(1) | 14(1) | -1(1) | -2(1) | -3(1) |
| C(7) | 17(1) | 17(1) | 14(1) | -1(1) | -1(1) | -1(1) |
| C(8) | 17(1) | 17(1) | 17(1) | 0(1) | -4(1) | -4(1) |
| C(9) | 21(1) | 24(1) | 19(1) | 2(1) | -4(1) | -4(1) |
| C(10) | 25(1) | 24(1) | 26(1) | 8(1) | -7(1) | -4(1) |
| C(11) | 27(1) | 21(1) | 34(1) | 2(1) | -11(1) | 1(1) |
| C(12) | 23(1) | 22(1) | 30(1) | -4(1) | -7(1) | 3(1) |
| C(13) | 19(1) | 18(1) | 20(1) | -4(1) | -5(1) | 0(1) |
| C(14) | 18(1) | 22(1) | 20(1) | -6(1) | -4(1) | 1(1) |
| C(15) | 18(1) | 27(1) | 14(1) | -1(1) | 3(1) | -3(1) |
| C(16) | 16(1) | 24(1) | 14(1) | -1(1) | 2(1) | -1(1) |
| C(17) | 30(1) | 31(1) | 17(1) | 4(1) | 4(1) | -2(1) |
| C(18) | 20(1) | 28(1) | 19(1) | -4(1) | -1(1) | -1(1) |

Table. Hydrogen coordinates ( x $10^4$) and isotropic displacement parameters ($Å^2$x $10^{3}$) for compound 21.

| | x | y | z | U(eq) |
|---|---|---|---|---|
| H(3) | 10057 | 1971 | 6209 | 25 |
| H(4) | 10466 | 2657 | 4206 | 23 |
| H(9) | 8225 | 5792 | 2382 | 26 |
| H(10) | 7254 | 7085 | 1874 | 30 |
| H(11) | 5682 | 7799 | 3285 | 33 |
| H(12) | 5067 | 7212 | 5213 | 30 |
| H(15A) | 6078 | 4089 | 7669 | 23 |
| H(15B) | 5305 | 4930 | 8103 | 23 |
| H(16) | 8671 | 4600 | 8442 | 22 |
| H(17A) | 8151 | 4301 | 10601 | 39 |
| H(17B) | 7278 | 3662 | 9651 | 39 |
| H(17C) | 6270 | 4379 | 10262 | 39 |
| H(18A) | 8772 | 5715 | 9838 | 34 |
| H(18B) | 6882 | 5852 | 9631 | 34 |
| H(18C) | 8032 | 6014 | 8487 | 34 |

Table. Torsion angles [°] for compound 21.

| | | | |
|---|---|---|---|
| C(14)-N(1)-C(1)-C(7) | 1.7(3) | C(13)-C(8)-C(9)-C(10) | 1.4(3) |
| C(15)-N(1)-C(1)-C(7) | 179.72(17) | C(7)-C(8)-C(9)-C(10) | -178.62(18) |
| C(14)-N(1)-C(1)-C(2) | -177.54(17) | C(8)-C(9)-C(10)-C(11) | -1.0(3) |
| C(15)-N(1)-C(1)-C(2) | 0.5(3) | C(9)-C(10)-C(11)-C(12) | -0.2(3) |
| N(1)-C(1)-C(2)-C(5) | 179.68(18) | C(10)-C(11)-C(12)-C(13) | 1.1(3) |
| C(7)-C(1)-C(2)-C(5) | 0.4(2) | C(11)-C(12)-C(13)-C(8) | -0.7(3) |
| N(1)-C(1)-C(2)-S(1) | -1.6(3) | C(11)-C(12)-C(13)-C(14) | 178.69(19) |
| C(7)-C(1)-C(2)-S(1) | 179.11(18) | C(9)-C(8)-C(13)-C(12) | -0.5(3) |
| C(3)-S(1)-C(2)-C(5) | 0.50(15) | C(7)-C(8)-C(13)-C(12) | 179.51(18) |
| C(3)-S(1)-C(2)-C(1) | -178.2(2) | C(9)-C(8)-C(13)-C(14) | -179.91(18) |
| C(2)-S(1)-C(3)-C(4) | -0.24(17) | C(7)-C(8)-C(13)-C(14) | 0.1(3) |
| S(1)-C(3)-C(4)-C(5) | -0.1(2) | C(1)-N(1)-C(14)-O(2) | 177.26(18) |
| C(1)-C(2)-C(5)-C(4) | 178.46(16) | C(15)-N(1)-C(14)-O(2) | -0.8(3) |
| S(1)-C(2)-C(5)-C(4) | -0.7(2) | C(1)-N(1)-C(14)-C(13) | -2.3(3) |
| C(1)-C(2)-C(5)-C(6) | -0.2(2) | C(15)-N(1)-C(14)-C(13) | 179.57(16) |
| S(1)-C(2)-C(5)-C(6) | -179.37(12) | C(12)-C(13)-C(14)-O(2) | 2.5(3) |
| C(3)-C(4)-C(5)-C(2) | 0.5(2) | C(8)-C(13)-C(14)-O(2) | -178.10(19) |
| C(3)-C(4)-C(5)-C(6) | 178.7(2) | C(12)-C(13)-C(14)-N(1) | -177.92(17) |
| C(2)-C(5)-C(6)-O(1) | 179.73(19) | C(8)-C(13)-C(14)-N(1) | 1.5(3) |
| C(4)-C(5)-C(6)-O(1) | 1.5(4) | C(1)-N(1)-C(15)-C(16) | 81.6(2) |
| C(2)-C(5)-C(6)-C(7) | 0.0(2) | C(14)-N(1)-C(15)-C(16) | -100.3(2) |
| C(4)-C(5)-C(6)-C(7) | -178.2(2) | N(1)-C(15)-C(16)-C(18) | 68.8(2) |
| N(1)-C(1)-C(7)-C(8) | 0.1(3) | N(1)-C(15)-C(16)-C(17) | -168.19(17) |
| C(2)-C(1)-C(7)-C(8) | 179.39(16) | | |
| N(1)-C(1)-C(7)-C(6) | -179.69(17) | | |
| C(2)-C(1)-C(7)-C(6) | -0.4(2) | | |
| O(1)-C(6)-C(7)-C(1) | -179.48(19) | | |
| C(5)-C(6)-C(7)-C(1) | 0.2(2) | | |
| O(1)-C(6)-C(7)-C(8) | 0.8(3) | | |
| C(5)-C(6)-C(7)-C(8) | -179.51(19) | | |
| C(1)-C(7)-C(8)-C(9) | 179.10(18) | | |
| C(6)-C(7)-C(8)-C(9) | -1.2(3) | | |
| C(1)-C(7)-C(8)-C(13) | -0.9(3) | | |
| C(6)-C(7)-C(8)-C(13) | 178.78(18) | | |

Table. Hydrogen bonds for compound 21 [Å and °].

| D-H...A | d(D-H) | d(H...A) | d(D...A) | <(DHA) |
|---|---|---|---|---|
| C(3)-H(3)...O(2)#1 | 0.95 | 2.35 | 3.067(3) | 132.4 |
| C(15)-H(15A)...S(1) | 0.99 | 2.74 | 3.357(2) | 120.9 |
| C(16)-H(16)...S(1) | 1.00 | 2.97 | 3.517(2) | 115.2 |

Symmetry transformations used to generate equivalent atoms:

#1 -x+3/2,y-1/2,-z+3/2

Figure SI-1. Fully-labeled ORTEP diagram of major lactone **12** with thermal ellipsoids drawn at the 50% probability level and H atoms drawn as arbitrary spheres.

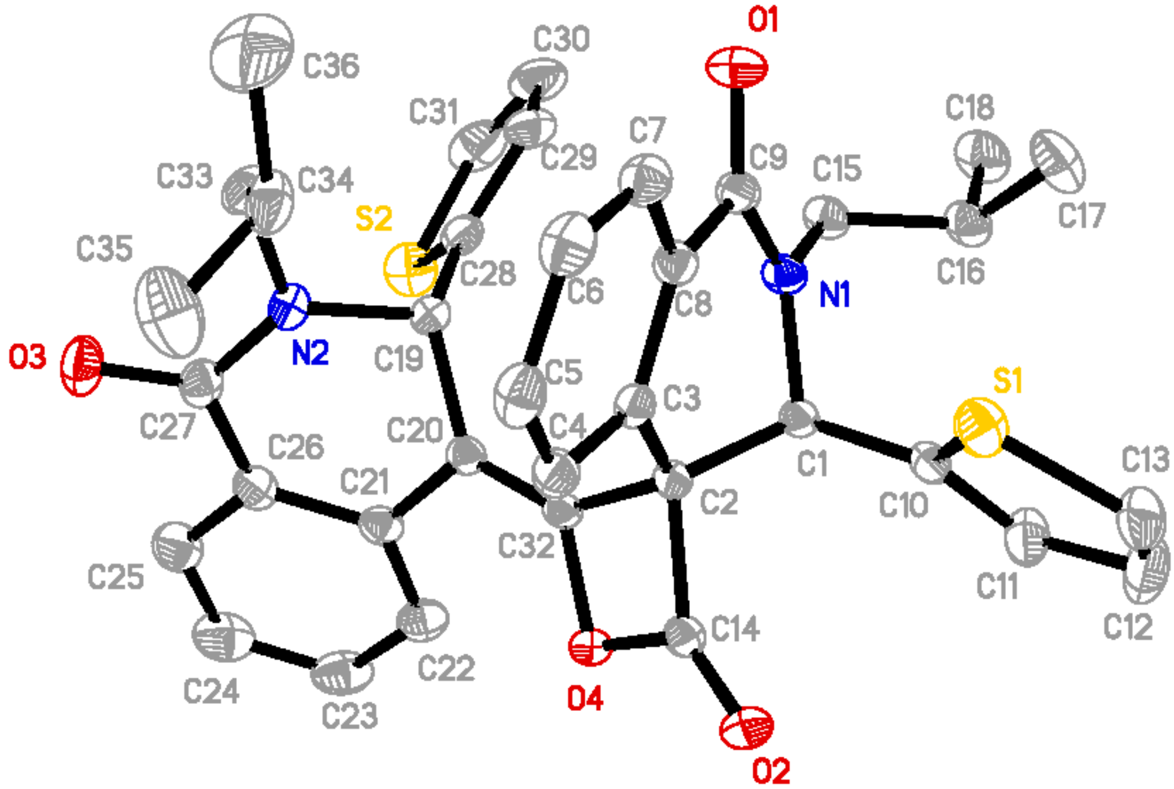

Figure SI-2. Fully-labeled ORTEP diagram of major lactone **21** with thermal ellipsoids drawn at the 50% probability level and H atoms drawn as arbitrary spheres.

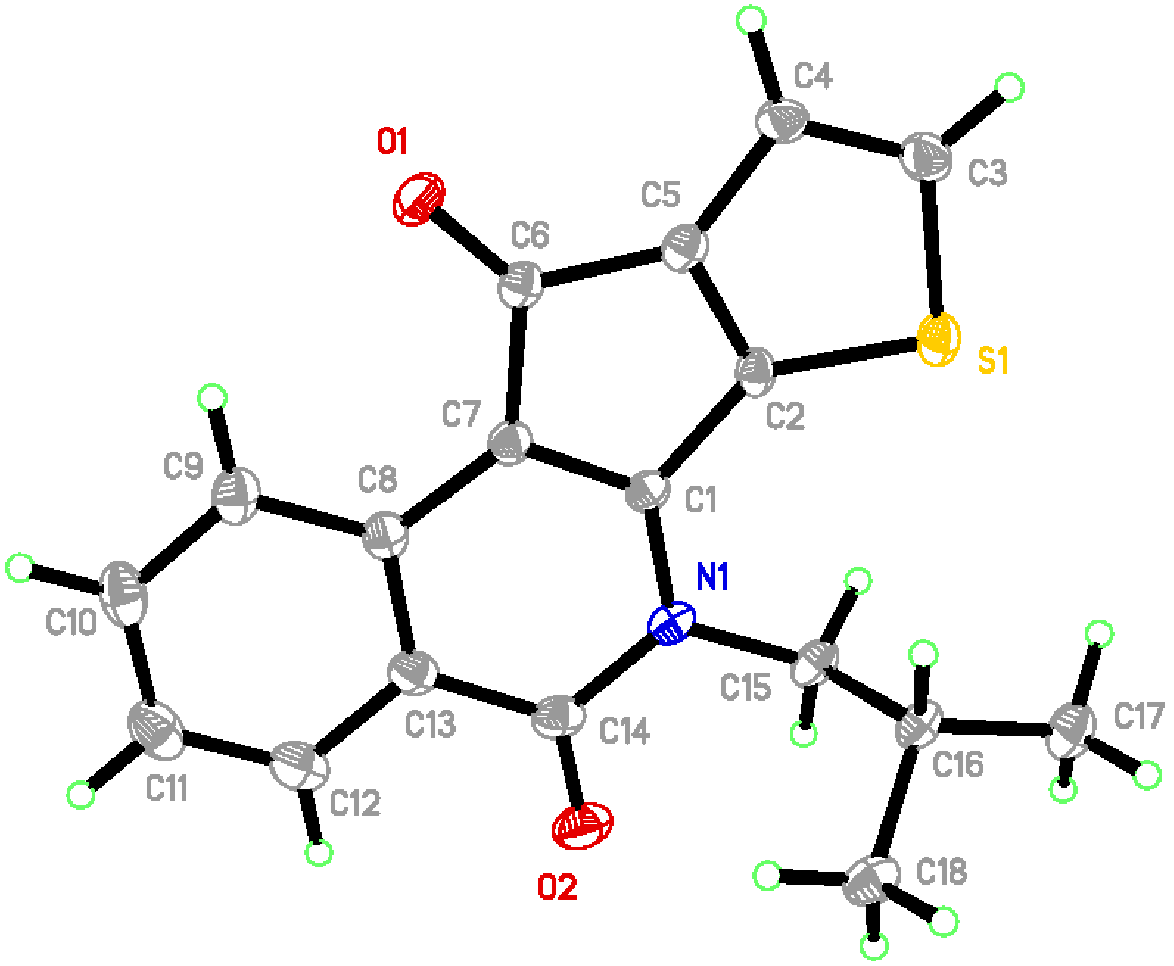

Supplement: Supplementary file 1 [file molecules-27-00066-s001.zip › molecules-1490123-supplementary.pdf]
